# Coherence properties of the high-energy fourth-generation X-ray synchrotron sources

**DOI:** 10.1107/S1600577519013079

**Published:** 2019-11-01

**Authors:** R. Khubbutdinov, A. P. Menushenkov, I. A. Vartanyants

**Affiliations:** a Deutsches Electronen-Synchrotron DESY, Notkestrasse 85, D-22607 Hamburg, Germany; b National Research Nuclear University MEPhI (Moscow Engineering Physics Institute), Kashirskoe shosse 31, Moscow 115409, Russian Federation

**Keywords:** diffraction-limited storage ring, energy spread, cross-spectral density function, coherent-mode representation, degree of coherence, coherent fraction

## Abstract

An analysis of the coherence properties of the fourth-generation high-energy storage rings with emittance values of 10 pm rad is performed. It is presently expected that a storage ring with these low emittance values will reach diffraction limit at hard X-rays. Simulations of coherence properties were performed with the *XRT* software and an analytical approach for different photon energies from 500 eV to 50 keV. It was demonstrated that a minimum photon emittance (diffraction limit) reached at such storage rings is λ/2π. Using mode decomposition it is shown that, for the parameters of the storage ring considered in this work, the diffraction limit will be reached for soft X-ray energies of 500 eV. About ten modes will contribute to the radiation field at 12 keV photon energy and even more modes give a contribution at higher photon energies. Energy spread effects of the electron beam in a low-emittance storage ring were analysed in detail. Simulations were performed at different relative energy spread values from zero to 2 × 10^−3^. A decrease of the degree of coherence with an increase of the relative energy spread value was observed. This analysis shows that, to reach the diffraction limit for high photon energies, electron beam emittance should go down to 1 pm rad and below.

## Abstract 2

Coherence properties of the fourth-generation high-energy storage rings with low-emittance values are discussed.

## 1. Introduction   

Recently it was realized that, due to a new conceptual approach, *i.e.* the multi-bend achromat synchrotron storage ring design, the brightness of next-generation X-ray storage rings may be increased by two to three orders of magnitude (Eriksson *et al.*, 2014 ▸; Hettel, 2014 ▸). This immediately implies that the coherent flux of these storage rings will be higher by two orders of magnitude as well and, by that, will be approaching the so-called diffraction limit. Next-generation synchrotron sources have the potential to make a great contribution to today’s major challenges in investigation of multi-functional hybrid materials, electronic transport phenomena and electrochemical processes in charge storage materials under working conditions, as well as materials under extreme conditions of pressure and temperature with highest resolution and sensitivity (Weckert, 2015 ▸).

The first storage ring constructed using multi-bend achromat technology was the 3 GeV synchrotron source MAX IV (Lund, Sweden), which recently reached its planned specifications of horizontal emittance of about 200–330 pm rad (depending on insertion device gap settings) (Tavares *et al.*, 2014 ▸). The high-energy ESRF 6 GeV storage ring is presently under reconstruction to the EBS ESRF facility (https://www.esrf.eu/home/UsersAndScience/Accelerators/ebs---extremely-brilliant-source/ebs-parameters.html) that is planned to reach a horizontal emittance of 133 pm rad. The Brazilian SIRIUS 3 GeV project is presently in the commissioning phase with horizontal emittance planned in the range 150–250 pm rad (Rodrigues *et al.*, 2018 ▸), and other facilities worldwide (APS-U, SPring-8, ALS, Soleil, Diamond, *etc*.) are in construction or at the planning stage. At DESY in Hamburg, Germany, an upgrade of the high-energy 6 GeV storage ring PETRA III to the PETRA IV facility is also planned (Schroer *et al.*, 2019 ▸). The world’s lowest emittance of about 10 pm rad for hard X-rays is targeted at this storage ring (Schroer *et al.*, 2018 ▸).

Source brilliance and coherence of the future storage rings are the keys for successful synchrotron radiation experiments. The high degree of coherence will allow the focusing of the synchrotron beams efficiently to the nanometre range without spatial filtering of flux (Singer & Vartanyants, 2014 ▸). It will allow an effective application of coherence-based techniques such as coherent diffraction imaging (CDI) potentially reaching sub-nanometre resolution (Schroer & Falkenberg, 2014 ▸). It will also extend photon correlation techniques into the regime of nanoseconds and allow for low dose correlation experiments (Shpyrko, 2014 ▸).

In order to reach all these goals, a better understanding of the coherence properties of radiation close to the diffraction limit is necessary. Ultimate storage rings are expected to have a high degree of coherence, which means that traditional methods of X-ray tracing will not be sufficient to predict parameters of X-ray beams at the experimental stations. Also, some intrinsic electron beam parameters such as an unavoidable energy spread of electrons in a storage ring may influence the coherence properties of X-ray beams.

It is interesting to note that even basic parameters of diffraction-limited sources are not well understood nowadays. For example, it is a long-standing debate on what is a correct asymptotic limit for the photon emittance of a diffraction-limited storage ring (Walker, 2019 ▸). It is commonly accepted that the diffraction-limited storage ring is the one with its electron beam emittance much lower than the natural emittance of single-electron radiation. For radiation described by the Gaussian functions, the diffraction-limited emittance is very well known from the uncertainty principle and is given by λ/4π, where λ is the wavelength of radiation (Kim, 1989 ▸). At the same time, several authors have demonstrated that the emittance of single-electron undulator radiation is given by λ/2π (Onuki & Elleaume, 2003 ▸; Tanaka & Kitamura, 2009 ▸). The problem is based on the fact that radiation from a single electron cannot be described by a Gaussian function. It is well known that in the far-field region and resonant conditions it is described by a sinc-function (Alferov *et al.*, 1973 ▸; Kim, 1986 ▸). Further on, we will carefully analyze this question and will give a definitive answer to this long-standing debate.^1^

Another important question is the influence of the energy spread of electrons in a storage ring on the coherence properties of the photon beams. It was first outlined by Tanaka & Kitamura (2009 ▸) that energy spread effects may affect the brightness of the X-ray storage rings. In a recent paper by Geloni *et al.* (2018 ▸), energy spread effects on brightness and coherence were carefully analysed for the low-emittance storage rings using an analytical approach. As a result of this analysis, it was shown that the brightness of diffraction-limited sources with small but finite emittance only slightly depends on the energy spread effects; the same is valid for the coherence function centred at the optical axis of the undulator with stronger effects at off-axis values. It was also demonstrated that the values of brightness obtained by this approach substantially differ from the results of Tanaka & Kitamura (2009 ▸). We will discuss in detail the effect of energy spread on the coherence properties of the low-emittance storage rings.

Presently it is a big demand to develope adequate and effective methods that may correctly describe properties of radiation from the ultimate storage rings close to the diffraction limit. Clearly, such a description should be based on the application of the first- and higher-order correlation functions (Mandel & Wolf, 1995 ▸; Vartanyants & Singer, 2016 ▸). Presently several codes have been developed to describe partial coherence radiation from conventional storage rings (see, for example, Chubar & Elleaume, 1998 ▸; Lee & Zhang, 2007 ▸; Shi *et al.*, 2014 ▸; Meng *et al.*, 2015 ▸). Unfortunately, these codes may be inefficient for simulating properties of diffraction-limited storage rings or require a substantial computer power and time to perform simulations. In this work for the analysis of coherence properties of the low-emittance storage rings, we used the recently developed computer code *XRT* (Klementiev & Chernikov, 2014 ▸) that supports parallel calculations on GPU and allows efficient simulation of all necessary correlation functions.

In this work, we analysed coherence properties of a high-energy low-emittance (10 pm rad and below) storage ring for different photon energies starting from soft X-rays of 500 eV up to hard X-rays of 50 keV. We introduced energy spread effects in our simulations and specifically examined the following values: zero energy spread value, 1 × 10^3^ and 2 × 10^3^. These studies may be of particular interest for the future PETRA IV facility with its record parameters (Schroer *et al.*, 2018 ▸).

The paper is organized as follows. In the next section, a short theoretical summary of the first-order correlation functions will be presented. In the same section, the basic principles of undulator radiation and its analytical description will be discussed. In the third and main section of the paper, simulations and results of the analysis of coherence properties of the low-emittance storage ring will be performed by different analysis tools. The paper will be finished with conclusions and outlook.

## 2. Theory   

### 2.1. Basics of coherence theory   

The measure of the first-order coherence is given by the mutual coherence function (MCF) defined as (Mandel & Wolf, 1995 ▸)
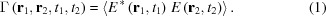
It describes correlations between two values of the electric field, 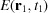 and 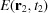, at different points 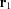 and 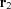 and times *t*_1_ and *t*_2_. The brackets 〈…〉 denote the ensemble average; for stationary sources the ensemble average coincides with the time average. We next introduce the cross-spectral density function (CSD) of a stationary source, which is obtained as the Fourier transform of the MCF (Mandel & Wolf, 1995 ▸),
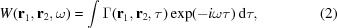
where 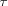 = 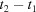. The spectral density of the radiation field is obtained when two points 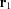 and 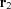 coincide, 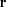 = 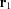 = 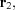


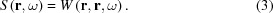
A convenient measure of spatial coherence is the normalized CSD,
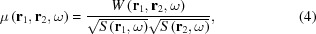
which is called the spectral degree of coherence (SDC). The values of this function, which are ranging from zero to one and depend on the pinhole separation 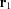 and 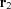, are determined in the classical Young’s experiment.

Another convenient measure of coherence is the global degree of coherence 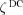, which characterizes the coherence properties of the wavefield by a single number and can be introduced as (Geloni *et al.*, 2008 ▸; Vartanyants & Singer, 2010 ▸)
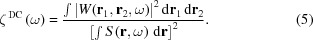
The values of the parameter 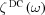 lie in the range 0 ≤ 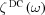 ≤ 1, where 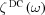 = 1 and 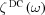 = 0 correspond to fully coherent and incoherent radiation, respectively.

In the quasi-monochromatic regime, it is possible to approximate the MCF in equation (1) as (Mandel & Wolf, 1995 ▸)
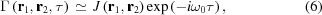
provided that 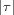 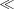 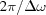, where 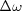 is the bandwidth of radiation. Here 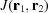 is the mutual optical intensity (MOI) defined as
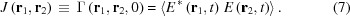
Taking into account all of the above, we may represent CSD for quasi-monochromatic radiation as
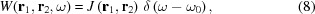
which describes correlations between two complex values of the electric field at different points 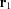 and 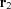 at a given frequency.

Finally, for quasi-monochromatic radiation, CSD 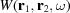 and MOI 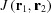 functions as well as spectral density 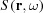 and intensity 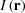 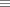 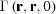 = 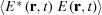 functions are equivalent.

### 2.2. Coherent-mode representation of the cross-spectral density function   

It is well known (Mandel & Wolf, 1995 ▸) that, under very general conditions, one can represent the CSD of a partially coherent, statistically stationary field of any state of coherence as a series
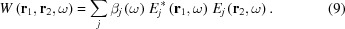
Here 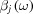 are eigenvalues and independent coherent modes 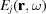 are eigen-functions of the Fredholm integral equation of the second kind,
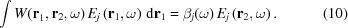
According to equations (3) and(9) the spectral density can be represented as
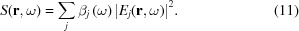
Substitution of equations (9) and (11) into equation (5) gives, for the global degree of coherence,
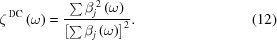
One has to remember important characteristics of this coherent mode decomposition: the mode functions 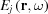 form an orthonormal set, the eigenvalues 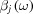 are real and non-negative, 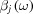 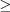 0 and 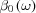 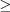 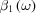 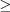 …. If there is only one single mode present then radiation is fully coherent. Thus we can define the coherent fraction (CF) of radiation 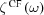 as an occupation or normalized weight of the first mode,
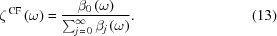
We will consider a quasi-monochromatic case and will omit frequency dependence in the following.

### 2.3. Gaussian Schell-model sources   

The Gaussian Schell-model (GSM) is a simplified but often used model (Vartanyants & Singer, 2010 ▸) that represents radiation from a real X-ray source based on the following approximations. The source is modelled as a plane two-dimensional source, the source is spatially uniform, *i.e.* the SDC depends only on the difference 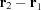, the SDC 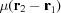 and spectral density 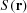 are Gaussian functions. In the frame of the GSM cross-spectral density function, spectral density and SDC are defined as (Mandel & Wolf, 1995 ▸)
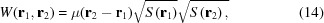


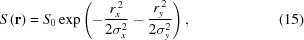


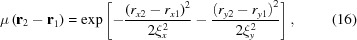
where 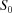 is a normalization constant, 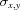 is the r.m.s. source size and 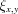 is the transverse coherence length in the source plane in the *x*- and *y*-direction, respectively. One of the important features of this model is that the CSD function is separable into two transverse directions,
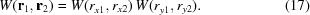
The same is valid for the global degree of coherence defined in equation (5),
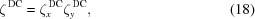
where in each transverse direction 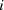 = *x,y* we have (Vartanyants & Singer, 2010 ▸)
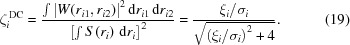
Coherent modes in the GSM are described by the Hermite–Gaussian functions (Gori, 1983 ▸; Starikov & Wolf, 1982 ▸; Vartanyants & Singer, 2010 ▸),
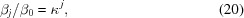


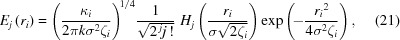
where the coefficient 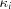 = 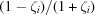 is introduced, 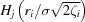 are the Hermite polynomials of order *j*, 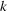 = 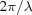 and 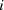 = 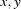. The zero mode is a Gaussian function and propagation of Hermite–Gaussian modes in the far-field region gives Hermite–Gaussian modes of the same shape. In the frame of GSM, according to equations (13) and (20) the coherent fraction of the radiation for one transverse direction may be determined as
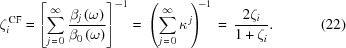



## 3. Synchrotron radiation from undulator sources   

### 3.1. Spectral brightness and phase space distribution   

The source may be completely characterized by its spectral brightness which is defined through the phase space distribution function that is a classical analogue of the Wigner distribution function (Wigner, 1932 ▸). According to this definition, the spectral brightness 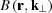 in the paraxial approximation is given by (Mandel & Wolf, 1995 ▸)
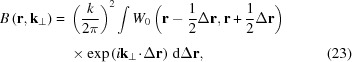
where 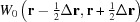 is the CSD function [equations (2) and (8)] defined at the source position, 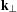 is a projection of the momentum vector 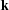 on the transverse plane, and the coordinates 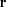 and 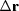 are introduced as 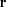 = 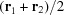 and 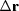 = 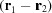.

In the synchrotron radiation community, it is conventional to define the distribution function in equation (23) in the phase space through the CSD function 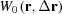 of the electric fields at the source position (Kim, 1986 ▸, 1989 ▸; Geloni *et al.*, 2015 ▸),
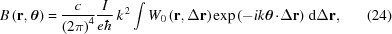
where **θ** is the angle between the optical axis and observation direction, *I* is the electron beam current, *e* is an electron charge, and 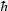 = 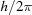 where *h* is Planck’s constant. This distribution taken at its maximum value, which typically for undulator radiation coincides with the optical axis for odd harmonics, is defined as the spectral brightness of the synchrotron source.

Approximating the far-field distribution of the electric field of undulator radiation in resonant conditions from a single electron by a Gaussian laser mode the following well known approximation for the brightness of undulator radiation was obtained (Kim, 1986 ▸, 1989 ▸),
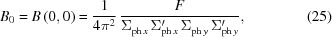
where *F* is the spectral photon flux into the central core. In equation (25), the total photon source size 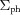 and divergence 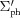 are defined as
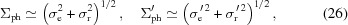
where 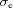 = 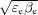 and 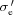 = 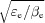 are the electron beam spatial size and angular divergence^2^, 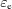 = 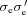 is the emittance of an electron beam at its waist, and 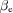 is the value of the betatron function in the centre of the undulator. Intrinsic characteristics of single-electron radiation in equation (26) were introduced by Kim (1989 ▸),
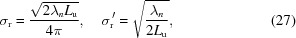
where 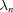 and 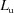 are the *n*th harmonic radiation wavelength and the undulator length. The total photon emittance of the undulator source is then introduced as
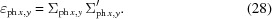
By that approach, the photon emittance 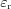 or photon phase space of a single electron in a Gaussian approximation is given by the value (Kim, 1989 ▸)
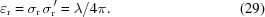
The diffraction-limited undulator source may be defined as the one with the electron beam parameters satisfying the following inequalities: 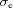 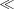 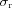 and 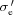 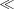 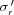. According to these definitions for the diffraction-limited source, the natural electron beam emittance has to be much smaller than the natural emittance of single-electron radiation, 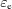 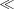 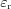 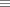 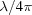.

### 3.2. Energy spread effects   

The energy spread of the electron beam is an energy distribution of electrons in the bunch, which for synchrotron radiation obeys Gaussian statistics. As noted in several publications (Onuki & Elleaume, 2003 ▸; Tanaka & Kitamura, 2009 ▸), energy spread effects may influence the properties of the synchrotron radiation source. By approximating the angular and spatial profile of the flux density by Gaussian functions for each transverse direction, the following expressions for the beam size and divergence were obtained (Tanaka & Kitamura, 2009 ▸),
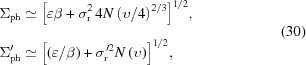
where 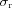 and 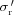 are intrinsic characteristics of single-electron radiation, defined in equations (27). A normalization factor 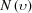 for the energy spread of the storage ring in equations (30) is defined as
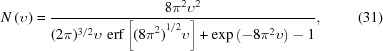
with 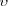 = 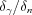 being the ratio between the relative energy spread value 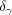 = 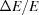 and the relative bandwidth 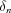 = 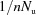 of the *n*th harmonic of an undulator with 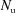 periods. The normalization function 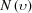 for zero relative energy spread is equal to one, 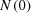 = 1. Note, that the source size for natural single-electron radiation in equation (30) is effectively two times larger than defined in equation (27). This difference originates from a non-Gaussian angular profile of far-field radiation from a single electron that is taken into account in equation (30). This also leads to a relaxed condition for the diffraction-limited source: 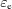 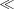 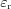 = 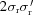 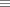 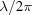. We will see in the following, by performing simulations, which of two conditions is satisfied in the limit of small electron beam emittance for the diffraction-limited undulator source.

In a recent paper by Geloni *et al.* (2018 ▸), energy spread effects on brightness and coherence were carefully analysed using directly equation (24) with an account of energy spread effects. For the far-field distribution of the electric field emitted by an electron with the energy deviating from the resonant photon energy of the undulator radiation harmonic, the following expression was used in equation (24) (Geloni *et al.*, 2018 ▸),
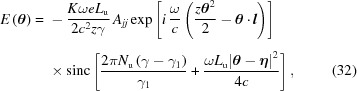
where γ and 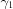 are the Lorentz factors at a given and resonant frequency electron energy, *z* is the distance from the centre of the undulator source, *K* is the maximum undulator deflection parameter, and the coupling parameter 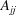 is defined as 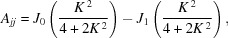
where 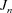 is the *n*th-order Bessel function of the first kind. In expression (32), energy offset 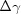 = γ − γ_1_, different entering angles **η** and various axis offset ***l*** of the electron in the undulator are explicitly taken into account.

In our further analysis, we will need the amplitude of the field at the source position. It is determined directly from expression (32) by applying the propagator in free space in paraxial approximation and is given by the following expression (Geloni *et al.*, 2015 ▸),
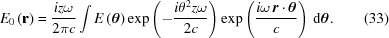
To obtain the source size and divergence for non-Gaussian field distributions we determined them by calculating the second moments or variances of the intensity distribution of the corresponding variables in each direction, respectively (Onuki & Elleaume, 2003 ▸),
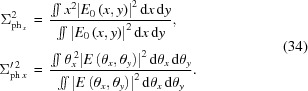
In addition to energy spread effects, the source size and source divergence are influenced by undulator detuning – a shift of the undulator harmonic wavelength λ_*n*_ relative to the selected radiation wavelength λ (Coisson, 1988 ▸). Detuning of the radiation wavelength from the resonant undulator harmonic value towards lower energies is very often used in practice to gain higher flux than at the resonant photon energy (Onuki & Elleaume, 2003 ▸). Depending on its value, detuning may have a stronger effect (a bigger linear and angular size variation) than energy spread but is out of the scope of the present paper. In this work, the radiation field was calculated in the monochromatic regime at a fixed resonance frequency. As such, no averaging over the frequency values was performed here.

### 3.3. Coherent fraction   

It is well established in the synchrotron radiation community that the coherent flux of a zero-emittance beam is given by 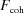 = 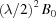 (see, for example, Kim, 1986 ▸; Geloni *et al.*, 2015 ▸). Importantly, this is an exact result that does not depend on the Gaussian approximation of single-electron radiation. Next, if we define the flux of the synchrotron source with certain values of emittance through brightness as in equation (25), we obtain
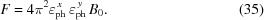
Strictly speaking, this expression is valid only for a Gaussian approximation of the electron-beam source parameters and natural single electron radiation with emittance values 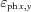 defined in equation (28) (see, for discussion, Geloni *et al.*, 2018 ▸). We will introduce the coherent fraction as
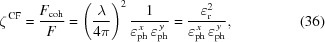
where 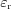 is the conventional emittance of single-electron radiation defined in equation (29). We will see in the following how expression (36) will be modified due to the non-Gaussian behaviour of single-electron radiation in the far-field region.

## 4. Results and discussion   

As an example of the diffraction-limited undulator source, we considered a high-energy storage ring operating at 6 GeV (for parameters of the source see Tables 1 ▸ and 2 ▸). For the electron emittance as a basic value, we considered 10 pm rad both in the vertical and horizontal direction by considering rather a round beam shape. We also analysed a broader range of electron emittance values from 1 pm rad to 300 pm rad to have a better understanding of the photon properties of the source. Simulations were performed either in the far-field region at the distance of 30 m from the source or directly at the source position that was considered in the middle of the undulator. Four energy values, 500 eV, 12 keV, 24 keV and 50 keV, were considered in this work. Comparing the natural emittance of single-electron radiation at these energies with the designed emittance of the source (see Table 2 ▸) we see that at the energy of 12 keV emittance values are comparable, at 500 eV, 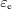 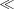 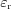, and at 24 keV as well as at 50 keV, 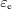 > 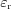. From that, we may expect to reach the diffraction limit at 500 eV and have parameters of radiation close to the diffraction-limited source at 12 keV. We also would expect that, at higher energies of 24 keV and 50 keV, radiation will be highly coherent but not diffraction-limited. All simulations were performed with the *XRT* software (Klementiev & Chernikov, 2014 ▸) (for details of the *XRT* software see the supporting information) and compared with the results of analytical calculations.

### 4.1. Photon emittance   

**Figure 1:**
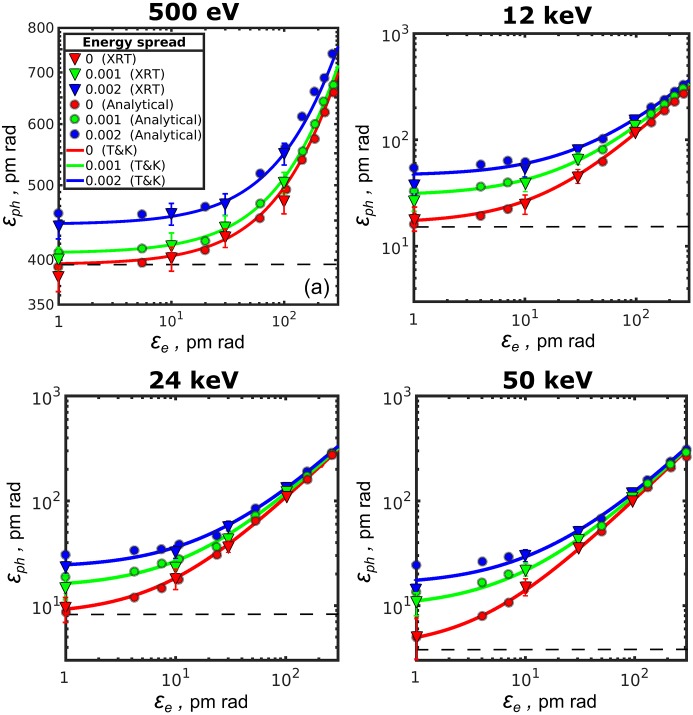
Photon emittance 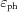 as a function of the electron beam emittance 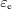 for the different values of the photon energy and energy spread in one transverse direction. Triangles are *XRT* simulations, circles are analytical calculations and lines are the values obtained from the Tanaka & Kitamura (2009 ▸) (T&K) approach [equations (30) and (31)]. Red, green and blue colour correspond to 0, 1 × 10^−3^ and 2 × 10^−3^ relative energy spread values, respectively. Note the different scale for the 500 eV emittance value. The dashed horizontal line corresponds to the value of the photon emittance of λ/2π.

Results of simulations performed by the *XRT* software of the photon emittance as a function of the electron beam emittance values from 1 pm rad to 300 pm rad for different relative energy spread values are presented in Fig. 1 ▸. X-ray radiation of the electron beam was simulated for the synchrotron storage ring with the parameters presented in Tables 1 ▸ and 2 ▸ (for details see the supporting information). The photon emittance (triangles in Fig. 1 ▸) was calculated as a product of the source size and divergence [see equation (28)] which were obtained as the variance values of the intensity distributions of the corresponding variables at the source position or in the far-field region according to equations (34).

As a result of these simulations, we can see that the lower the electron emittance 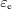, the lower the photon emittance 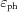. Importantly, we can observe that at 500 eV and 12 keV photon energies and at 10 pm rad electron beam emittance the photon emittance reaches its asymptotic value (note also the different scale for 500 eV photon energy). This is a clear indication that at these energies the synchrotron source may be considered as diffraction limited. We also observe that, at larger energy spread values and the same electron emittance, photon emittance is also increasing. However, the energy-spread-induced difference does not exceed 12% at 500 eV/10 pm rad, while at higher energies this difference goes up to 50%. This is due to the fact that at low photon energies the properties of the beam (source size and divergence) are comparably large, and their small changes caused by the energy spread effect are not noticeable. In contrast, the energy spread effects are revealed strongly at high energies due to smaller parameters of the radiation at these energies.

We compared these results with the analytical ones obtained using equations (32)–(34) (circles in Fig. 1 ▸) (see for details the supporting information). We see that the results of the analytical approach and simulations performed with the *XRT* software correspond to each other very well (both for different electron emittance values and for different energy spread values) within the margins of the error bars. Both approaches were also compared with the calculations made according to the approach of Tanaka & Kitamura (2009 ▸), equations (30)–(31) (shown by lines in Fig. 1 ▸). We observe that all three approaches give similar results for the three values of energy spread that were considered here.

As can be clearly seen in Fig. 1 ▸ (see also Table 3 ▸), the lowest value of the photon emittance for zero energy spread in our simulations is asymptotically reaching the value of 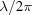, when electron emittance values are becoming sufficiently small. This is a strong indication that for low-emittance storage rings X-ray radiation cannot be approximated as Gaussian, because in this case the lowest photon emittance should reach the value of 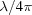 as suggested by Kim (1989 ▸). At the same time, these results are in concordance with the other results (see, for example, Onuki & Elleaume, 2003 ▸; Tanaka & Kitamura, 2009 ▸), where non-Gaussian behaviour of synchrotron radiation of a single electron was analysed.

Now we would like to determine a coherent fraction of radiation at different electron emittance values. If we use the conventional expression (36) with the emittance values 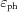 shown in Fig. 1 ▸ our coherent fraction values would reach an asymptotic value of 0.5 and would never reach 1. We slightly redefined our previous expression to the new one (see also Onuki & Elleaume, 2003 ▸),
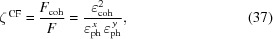
where 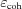 = 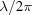 and 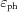 are photon emittance values obtained through different simulations. We would like also to note here that definitions of the coherent fraction through equations (13) and (37) are, in fact, equivalent. Indeed, the total flux of radiation *F* may be represented through the spectral density integrated over the solid angle (see Mandel & Wolf, 1995 ▸). The spectral density may be decomposed into a sum of modes, which are orthonormal. Performing angular integration in this expression will lead to a sum of mode eigenvalues. Following the same arguments, we may represent the coherent flux *F*_coh_ as a weight of the zero mode.

**Figure 2:**
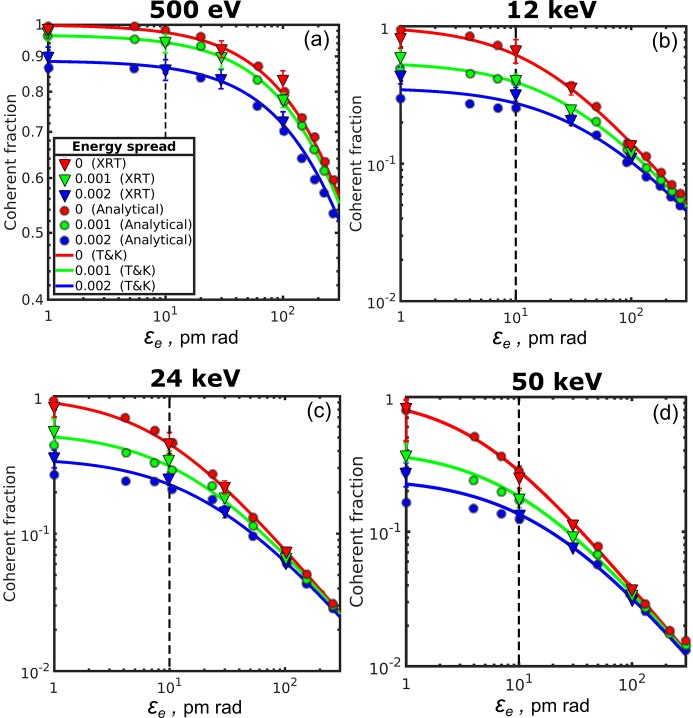
Coherent fraction of radiation 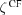 as a function of the electron beam emittance 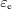 for the different values of the photon energy and energy spread in one transverse direction calculated according to equation (37). Triangles are *XRT* simulations, circles are analytical calculations, and lines are the values obtained by the Tanaka & Kitamura (2009 ▸) (T&K) approach [equations (30)–(31)]. Red, green and blue colour correspond to 0, 1 × 10^−3^ and 2 × 10^−3^ relative energy spread values, respectively. Note the different scale for 500 eV coherent fraction value. Dashed vertical lines correspond to the value of the electron emittance of 10 pm rad.

The results of our simulations in one transverse direction are presented in Fig. 2 ▸. First, we determined the coherent fraction from *XRT* simulations (triangles) by using expression (37) and the results of emittance simulations shown in Fig. 1 ▸. Then we compared the results of *XRT* simulations with the analytical calculation. Expressions (32) and (33) were used to calculate the wavefield amplitudes in the far-field region and at the source position. Equations (34) were applied to determine the source parameters. Using the same expression (37) we obtained analytical values (circles) of the coherent fraction shown in Fig. 2 ▸. Finally, we used in the definition of the coherent fraction [equation (37)] the values provided by equations (30) and (31) (Tanaka & Kitamura, 2009) (shown by lines).

We see that all three results excellently agree with each other and show the same trend that was just discussed for emittance. It is important to note that to obtain this result we have to use expression (37) instead of the commonly used equation (36). Next, we will turn to a more general definition of coherent fraction through coherent mode decomposition.

### 4.2. Representation of the cross-spectral density by the coherent mode decomposition   

As discussed in the *Theory* section[Sec sec2], the four-dimensional CSD function may be decomposed to a sum of two-dimensional coherent modes as given in equation (9). The *XRT* software is providing an opportunity to directly analyze these modes (see the supporting information for details).

**Figure 3:**
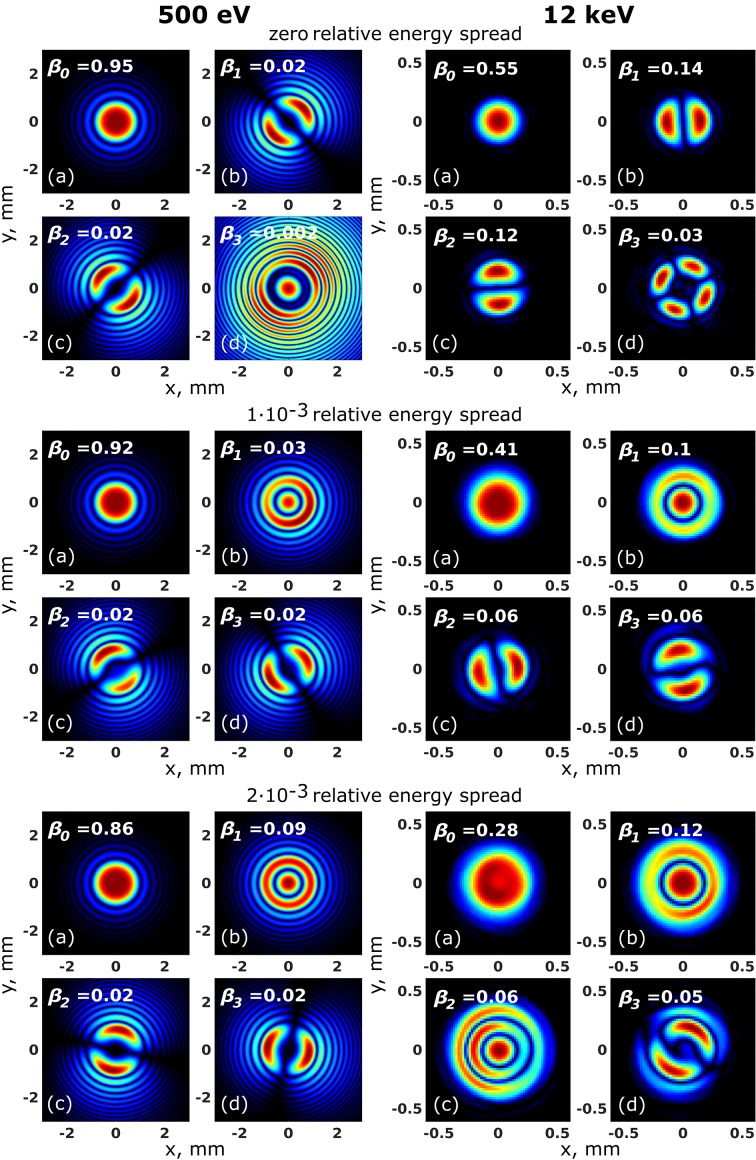
First four modes and their normalized weights 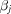 obtained from the coherent mode decomposition of the CSD at 500 eV (left column) and 12 keV (right column) photon energy for three different relative energy spread values obtained by *XRT* simulations.

We performed mode decomposition of the CSD according to equations (9)–(11) using the *XRT* software. We performed our analysis for the studied case of 10 pm rad electron beam emittance. The mode decomposition was used to determine the shape and contribution of each mode at different photon energies and at different values of relative energy spread. The first four modes and their normalized weights for 500 eV and 12 keV photon energy are shown in Fig. 3 ▸ (results of simulations for 24 keV and 50 keV are given in Fig. S2 of the supporting information). An orthogonal set of modes determined by our simulations, for the whole range of energy spread values from 0 to 2 × 10^−3^, represents a mixture of Laguerre–Gaussian-like and Hermite–Gaussian-like modes (Siegman, 1986 ▸). These two different sets of modes have different symmetry: Hermite–Gaussian modes may be represented as a product of two separable amplitude functions in the transverse plane and, contrary to that, Laguerre–Gaussian modes possess cylindrical symmetry and do not allow factorization in two orthogonal directions. It can also be noticed that the contribution of Laguerre–Gaussian modes is increased for both energies with the increase of the energy spread values. It is interesting to note here that the contribution of Laguerre–Gaussian modes is coming solely from the energy spread effect. It can be shown that if all electron offset parameters (entering angle **η** and axis offset ***l***) are put to zero then all modes of radiation field may be represented by Laguerre–Gaussian modes. This all means that, in general, for the diffraction-limited source, it is not possible to factorize the CSD function in two orthogonal transverse directions and define the degree of coherence as a product of its values in each direction [see equation (18)].

**Figure 4:**
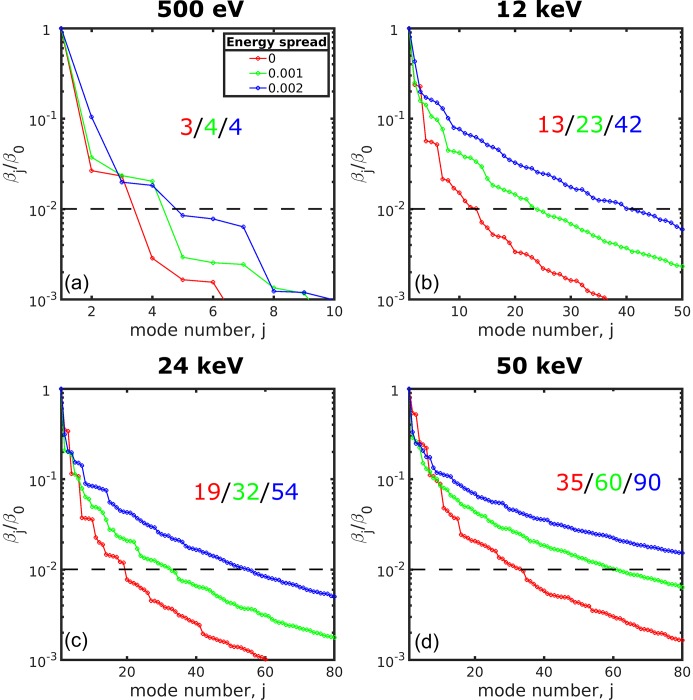
Weights of different modes normalized to the weight of a zero-mode 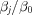 as a function of the mode number *j* for the different values of the photon energy and energy spread. The horizontal dashed lines correspond to the value of 1%. Points are connected by lines for better visibility. Red, green and blue colour correspond to 0, 1 × 10^−3^ and 2 × 10^−3^ relative energy spread values, respectively. The number of modes exceeding 1% threshold is given in each panel.

We also estimated the number of modes that contribute dominantly to the CSD [equation (9) and their spectral density, equation (11)]. We evaluated the weights of different modes normalized to zero mode 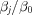 and introduced a threshold value of 1%. The values of these weights are presented in Fig. 4 ▸ as a function of the mode number for all photon energies and energy spread values considered in this work. As can be seen from this figure for 500 eV photon energy and zero energy spread only three modes contribute significantly and this value is increased only to four modes as energy spread values are increased. Already for 12 keV photon energy, the number of modes with a contribution higher than 1% is about ten. This rises with the increased energy spread values reaching 42 modes at 2 × 10^−3^ relative energy spread value. The number of modes that contribute dominantly to the CSD and spectral density with a threshold of 1% is 19 for 24 keV and 35 for 50 keV in the zero relative energy spread case. With the increase of energy spread to the value of 2 × 10^−3^ the number of modes contributing dominantly to the CSD is also increasing to 54 at 24 keV and to 90 at 50 keV, which is significantly larger than in the previous case of lower photon energies.

As soon as all mode weights were determined by the *XRT* software, we also determined the global degree of coherence for the considered photon energies and energy spread values according to equation (12) (see Table 4 ▸). The values of the global degree of coherence vary from 90% to 11% for photon energies from 500 eV to 50 keV, respectively. They drop down significantly with the increase of energy spread value for the same range of photon energies.

### 4.3. Coherent fraction of radiation   

**Figure 5:**
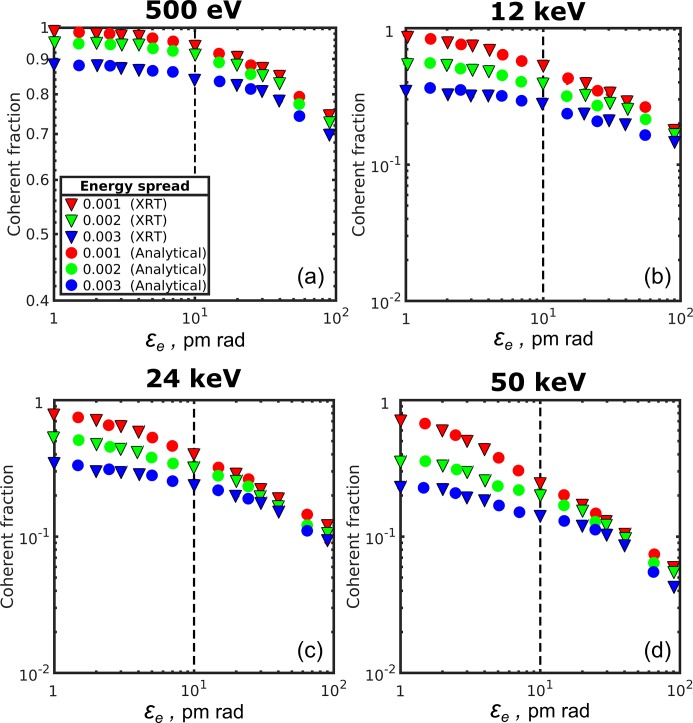
Coherent fraction of radiation 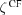 as a function of the electron beam emittance 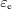 for the different values of the photon energy and energy spread in one transverse direction. Triangles are *XRT* simulations, circles are analytical calculations performed according to equation (13). Red, green and blue colour correspond to 0, 1 × 10^−3^ and 2 × 10^−3^ relative energy spread values, respectively. Dashed vertical line corresponds to the value of the electron emittance of 10 pm rad. Note the different scale for 500 eV coherent fraction value.

As soon as the mode values are normalized by the sum of all modes 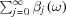 (as it is implemented in the *XRT* software), the value of the first mode naturally gives the coherent fraction of radiation [see equation (13)]. The values of the coherent fraction are presented in Fig. 5 ▸ for all photon energies considered in this work (shown by triangles) as a function of electron beam emittance for different values of energy spread. We can see from this figure (see also Table 4 ▸) that at 10 pm rad we are getting very high coherence values of about 95% at 500 eV and 55% at 12 keV at zero energy spread. With the increase of the energy spread these values become slightly lower at the energy spread values of 1 × 10^−3^ (91% and 41%) and significantly lower at the energy spread values of 2 × 10^−3^ (85% and 28%). At higher photon energies the coherent fraction of the radiation drops from 40% to 26% at 24 keV and from 26% to 15% at 50 keV, while energy spread increases from zero to 2 × 10^−3^.

We compared these results of *XRT* simulations with calculations of coherent fraction in the frame of the analytical approach (shown by circles in Fig. 5 ▸). Analytical simulations were performed by taking field amplitudes at the source and in the far-field region according to equations (32)–(33) and performing mode decomposition similar to the *XRT* code (see the supporting information for details). We see from Fig. 5 ▸ that the results of the analytical approach fit well to the results of the *XRT* simulations for all energies and energy spread values considered in this work.

### 4.4. Cross-spectral density function analyzed in one transverse direction   

Finally, we would like to compare the results obtained in the previous sections with the results of simulations of correlation functions in one direction. The coherent-mode representation of correlation functions, being very general, is providing an excellent theoretical insight into the problem. At the same time, the shape of the coherent modes is not easy to determine experimentally. Since the cross-spectral density 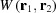 is a four-dimensional function, its determination from the experimental data is not a simple task. In most of the experiments (such as Young’s double pinhole experiment), correlation functions are determined in each direction separately (see, for example, Vartanyants *et al.*, 2011 ▸; Singer *et al.*, 2012 ▸; Skopintsev *et al.*, 2014 ▸). Next, it is assumed that the CSD of the whole field may be represented as a product of its orthogonal directions [see equation (17)]. We analysed this question for the case of the diffraction-limited source.

**Figure 6:**
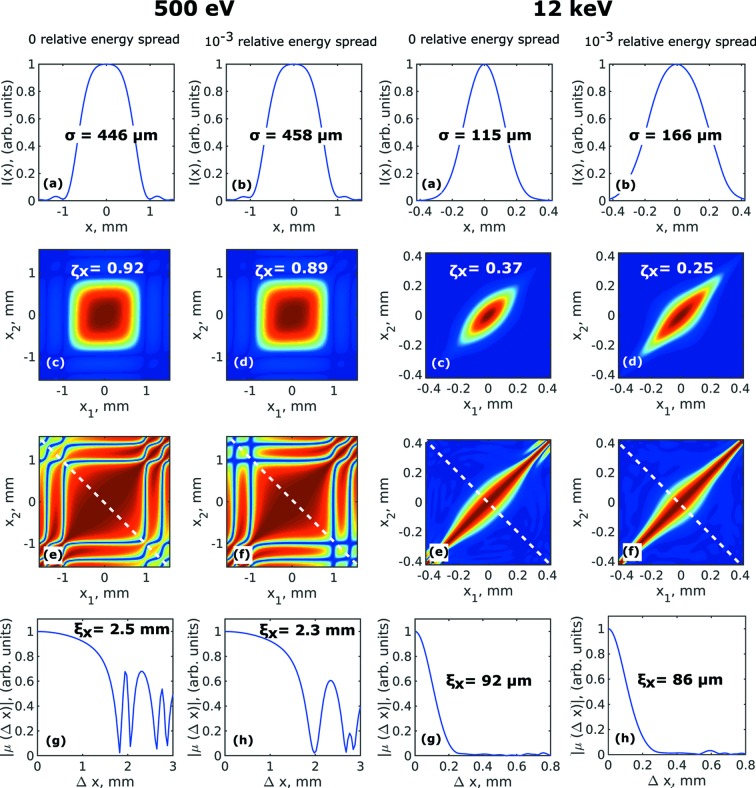
Simulations of the correlation functions in the horizontal direction performed by the *XRT* software for 500 eV (left column) and 12 keV (right column) photon energy. Intensity distribution *I*(*x*) (*a*, *b*), absolute value of the cross-spectral density in the horizontal direction |*W*(*x*_1_,*x*_2_)| (*c*, *d*), absolute value of the SDC |μ(*x*_1_,*x*_2_)| (*e*, *f*), and absolute value of the spectral degree of coherence along the anti-diagonal line [shown in (*e*, *f*)] as a function of separation of two points |μ(*Δx*)| (*g*, *h*) simulated in the horizontal direction 30 m downstream from the undulator source. In (*a*, *b*) σ is the r.m.s. value of the beam size, in (*c*, *d*) 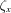 is the transverse degree of coherence, and in (*g*, *h*) 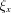 is the coherence length determined in the horizontal direction.

Simulations of the correlation functions in one transverse direction were performed in the far-field region at 30 m distance from the source. To determine these functions the *XRT* software and analytical expression of the wavefield from a single electron in the far-field region [see equation (32)] were used. The results of these simulations for two photon energies of 500 eV and 12 keV are presented in Table 5 ▸ and Fig. 6 ▸ (results of the analytical approach for the same energies are shown in Figs. S5–S6 of the supporting information). The intensity distribution *I*(*x,y*) [Figs. 6(*a*) and 6(*b*) ▸], absolute value of the CSD in the horizontal direction |*W*(*x*_1_,*x*_2_)| [Figs. 6(*c*) and 6(*d*) ▸], absolute value of the SDC |μ(*x*_1_,*x*_2_)| [Figs. 6(*e*) and 6(*f*) ▸], and absolute value of the spectral degree of coherence as a function of spatial separation of two points |μ(Δ*x*)| [Figs. 6(*g*) and 6(*h*) ▸] for both energies are presented in Fig. 6 ▸. It is easy to see from this figure that the functional dependence of these parameters is non-Gaussian for the 500 eV photon energy. But already at 12 keV these parameters can be safely described by Gaussian functions (the same is valid for higher energies). As a general rule, the more modes contribute to the CSD, the more this dependence resembles Gaussian. We determined the root mean square (r.m.s.) values 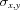 of the intensity distribution in the far-field region using equations (34) [see Figs. 6(*a*) and 6(*b*) ▸]. The values of the transverse degree of coherence 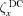 [see Figs. 6(*c*) and 6(*d*) ▸] were calculated according to equation (5). Anti-diagonal cuts of the spectral degree of the coherence function [see Figs. 6(*e*) and 6(*f*) ▸] were used to determine the coherence length of radiation 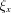 as its r.m.s. values according to equations (34). As can be seen from Fig. 6 ▸, results of simulations performed by the *XRT* software match extremely well to the ones performed analytically (see Figs. S5–S6 of the supporting information).

As can be seen from our simulations, the CSD function 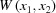 has a rectangular shape [see Figs. 6(*c*) and 6(*d*) ▸] because the coherence length of the beam is larger than the beam size at 500 eV. This is similar to our earlier observations at the X-ray free-electron laser source (Gorobtsov *et al.*, 2018 ▸). Also, in this diffraction-limited case, the SDC shows strong oscillations at the tails of the beam profile due to the fact that the total photon radiation is defined mostly by characteristics of single-electron radiation. This is also a reason why a Gaussian approximation is not valid in this diffraction-limited case and more careful analysis is required. Similar to our previous studies we also observed a decrease of the values of the transverse degree of coherence with the increase of energy spread (see Table 5 ▸). Similar simulations were performed for higher photon energies of 24 keV and 50 keV, and are summarized in Figs. S3–S4 of the supporting information and in Table 5 ▸.

Note that although the Gaussian approximation fits nicely for the spectral degree of coherence profile as well as for cross-spectral density at higher energies, the global degree of coherence is not equal to the product of transverse coherence values, 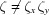 (compare Tables 4 ▸ and 5 ▸). This leads to the conclusion that correlation functions for synchrotron radiation close to the diffraction limit cannot be factorized in the two transverse directions.

## 5. Conclusions and outlook   

In summary, we have provided a detailed analysis of the coherence properties of a high-energy synchrotron storage ring with ultra-low emittance values near 10 pm rad and a wide range of photon energies from 500 eV to 50 keV. Such low values of electron beam emittance are expected to be reached at the PETRA IV facility (Schroer *et al.*, 2018 ▸, 2019 ▸). In addition, we analyzed the effect of electron energy spread on radiation properties of a low-emittance synchrotron ring for the same energy range. All simulations were performed using *XRT* software and were additionally compared with the results of analytical simulations based on equations of synchrotron radiation and also with approximated formulas given by Tanaka & Kitamura (2009 ▸). We note that all three approaches produced similar results for the whole set of parameters investigated in this work.

The following important lessons were learned during this work. In order to determine the properties of radiation from diffraction-limited sources, an approach based on statistical optics (Goodman, 1985 ▸; Mandel & Wolf, 1995 ▸) should be used^3^. For low-emittance storage rings, the radiation field can no longer be approximated by Gaussian functions. The single electron radiation distribution defines the beam profile in this low-emittance regime. As a consequence, a minimum photon emittance (diffraction limit) that may be reached on such storage rings is rather λ/2π than λ/4π, typical for Gaussian beams. As a result, the degree of coherence of the radiation goes to its asymptotic limit and approaches unity when the photon emittance is reaching the value of λ/2π.

Another lesson is that even for such low emittance values as 10 pm rad the true diffraction limit will be reached; in fact, only at soft X-ray energies of about 500 eV. In this case, only a few modes will contribute to the radiation field, but already at 12 keV the radiation field will consist of about ten modes and will start to be more Gaussian-like. This effect will be even more pronounced at high energies. In order to reach the true diffraction limit for hard X-rays, the emittance should be pushed down to about 1 pm rad, that is, unfortunately, out of reach for present technology.

Our results also show that correlation functions, describing radiation field properties such as the degree of coherence, cannot be factorized into two transverse directions for these low-emittance sources. For a full description of the radiation properties of these sources, eigenvalue decomposition of the radiation field has to be performed which offers good theoretical insight as well as complete generality. It also means that present experimental approaches which measure coherence properties of radiation in each direction separately (Vartanyants *et al.*, 2011 ▸; Singer *et al.*, 2012 ▸; Skopintsev *et al.*, 2014 ▸) should be generalized to 2D methods of coherence determination.

Another outcome of our work is the analysis of the electron energy spread effects on coherence properties of low-emittance storage rings. We demonstrate that this effect becomes more noticeable for low electron beam emittance. The larger the energy spread values, the more the source size and divergence are affected, and, as a consequence, the degree of coherence and coherent fraction value of the radiation are decreased. We found that, in order to keep high coherence values of radiation, the relative energy spread should not exceed the value of 1 × 10^−3^ at the electron emittance values of 10 pm rad. We should note that at 1 pm rad emittance values the energy spread should be sufficiently smaller than 1 × 10^−3^ to keep high coherence of the X-ray beam.

Finally, our results demonstrate that the coherence properties of the future diffraction-limited sources will be outstanding (Hettel, 2014 ▸; Weckert, 2015 ▸). We hope that the general approach and new tools for an adequate description of the coherence properties of synchrotron sources, provided in this work, will be helpful for the design and planning of future diffraction-limited sources worldwide.

## 6. Related literature   

The following references, not cited in the main body of the paper, have been cited in the supporting information: Jackson (1962 ▸); Sanchez del Rio (2018 ▸).

## Supplementary Material

In the supporting information file the details of XRT and analytical simulations are provided. DOI: 10.1107/S1600577519013079/pp5149sup1.pdf

## Figures and Tables

**Table 1:** Basic simulation parameters of the accelerator and undulator source for all photon energies

Electron energy	6.0 GeV
Beam current	100 mA
Horizontal and vertical electron beam emittance	10 pm rad
Horizontal and vertical betatron functions, β_*x*_, β_*y*_	2.0 m
Relative energy spread values	0; 1 × 10^−3^; 2 × 10^−3^
Undulator length	5 m

**Table 2:** Phase space parameters (in Gaussian approximation) of an X-ray source at different photon energies for 10 pm rad electron beam emittance and zero energy spread value

Photon energy (keV)	0.5	12	24	50
Number of undulator periods, *N*_u_	72	170	170	170
Number of harmonics, *n*	1st	3rd	3rd	5th
Single electron radiation size, σ_r_ (µm) [equation (27)]	12.5	2.55	1.79	1.25
Single electron radiation divergence, 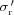 (µrad) [equation (27)]	15.7	3.2	2.3	1.5
Single electron radiation emittance, ∊_r_ = λ/4π (pm rad)	197	8.2	4.1	1.9
Electron beam size, σ_e_ (µm)	4.47	4.47	4.47	4.47
Electron beam divergence, 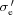 (µrad)	2.24	2.24	2.24	2.24
Total photon emittance, ∊_ph_ (pm rad) [equation (26)]	211	20	15.5	12.5

**Table 3:** Photon emittance values determined at different energies and zero electron energy spread using *XRT* simulations, an analytical approach and equations (30)–(31) Results are presented for the 10 pm rad and 1 pm rad electron emittance values.

Photon energy (keV)	0.5	12	24	50
∊_coh_ = λ/2π (pm rad)	395	16.4	8.2	3.9

10 pm rad natural electron emittance				
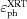 (pm rad)	398	24.6	18.5	15.2
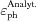 (pm rad)	402	24.9	17.7	13.8
∊_ph_^T&K^ (pm rad)	403	26.5	18.3	13.8
Source size (FWHM), 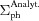 (µm)	59	15.9	13.5	12
Source divergence (FWHM), 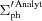 (µm)	37	9.3	7.5	6.5

1 pm rad natural electron emittance				
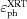 (pm rad)	380	20	10	5
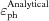 (pm rad)	391	16.3	8.9	5
∊_ph_^T&K^ (pm rad)	396	17.5	9.3	5
Source size (FWHM), 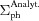 (µm)	58.6	12.4	9.1	6.7
Source divergence (FWHM), 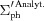 (µm)	37.5	7.8	5.7	4.1

**Table 4:** Global degree of coherence [equation (12)] and the coherent fraction [equation (13)] determined from coherent mode decomposition using *XRT* software and the analytical approach [equations (32) and (33)] All simulations were performed at the electron beam emittance value of 10 pm rad for different photon energies and energy spread values.

Photon energy (keV)	0.5	12	24	50
Zero relative energy spread				
Global degree of coherence, 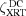	0.90	0.34	0.20	0.11
Coherent fraction, 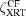	0.95	0.55	0.40	0.26
Coherent fraction, 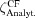	0.95	0.56	0.41	0.26

1 × 10^−3^ relative energy spread				
Global degree of coherence, 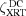	0.84	0.20	0.13	0.06
Coherent fraction, 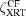	0.91	0.41	0.35	0.22
Coherent fraction, 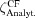	0.93	0.37	0.3	0.17

2 × 10^−3^ relative energy spread				
Global degree of coherence, 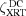	0.74	0.11	0.07	0.03
Coherent fraction, 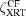	0.85	0.28	0.26	0.15
Coherent fraction, 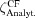	0.89	0.26	0.21	0.12

**Table 5:** Degree of coherence in one transverse direction obtained from the *XRT* simulations at 10 pm rad electron beam emittance compared with the analytical analysis for the different photon energies and relative energy spread values

Photon energy (keV)	0.5	12	24	50
Zero relative energy spread				
Degree of coherence, 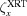	0.92	0.37	0.26	0.15
Degree of coherence, 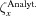	0.92	0.39	0.25	0.15
Degree of coherence, 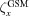	0.87	0.39	0.28	0.16

1 × 10^−3^ relative energy spread				
Degree of coherence, 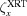	0.89	0.25	0.17	0.11
Degree of coherence, 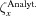	0.89	0.23	0.17	0.09
Degree of coherence, 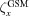	0.85	0.29	0.19	0.11

2 × 10^−3^relative energy spread				
Degree of coherence, 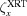	0.82	0.17	0.12	0.07
Degree of coherence, 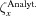	0.80	0.15	0.12	0.07
Degree of coherence, 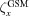	0.84	0.21	0.14	0.09

## Footnotes

1 When this work was finished the paper written by Walker (Walker, 2019 ▸) was published. The thorough analysis presented by the author was based on the synchrotron radiation simulations presented in the book by Onuki & Elleaume (2003 ▸). The author also discussed the question of diffraction limit and came to a similar conclusion as in our work for the so-called projected values of emittance.

2 This expression for the electron beam spatial size and angular divergence is valid when electron beam dispersion is neglected in a straight section.

3 As shown in our work, the Gaussian Schell-model, discussed earlier, should be used with caution for diffraction-limited sources.
