# Supplementary material for: Coherence properties of the high-energy fourth-generation X-ray synchrotron sources
Source: J Synchrotron Radiat. 2019 Nov 1;26(Pt 6):1851–62. doi: 10.1107/S1600577519013079 (PMC6853378; doi:10.1107/S1600577519013079)
Supplement: Supplementary file 1 [file s-26-01851-sup1.pdf]

# Supporting information for:

# Coherence properties of the high-energy fourth-generation X-ray synchrotron sources

R. Khubbutdinov[1,2], A. P. Menushenkov[2], and I. A. Vartanyants [1,2,*]

*[1]Deutsches Elektronen-Synchrotron DESY, Notkestrasse 85, D-22607 Hamburg, Germany*

*[2]National Research Nuclear University MEPhI (Moscow Engineering Physics Institute) Kashirskoe shosse 31, 115409 Moscow, Russia*

October 21, 2019

## S1. Details of XRT simulations

*a) Field amplitude and source parameters simulation*

To fully characterize source properties one needs to know the amplitude and intensity distributions at the source position and in the far field region, which were simulated at first by means of XRT software. The initial step was to determine synchrotron facility parameters as well as parameters of the undulator according to Table 1. The XRT software calculates an amplitude of the radiation for a single electron from the undulator source at the given photon energy (Jackson, 1962) on a given angular mesh (see Table S1). The transverse field from each electron gets individual random angular and coordinate offsets within the emittance distribution assuming Gaussian statistics of the electron bunch. An individual random shift to gamma Lorentz factor within the energy spread is counted as well (Klementiev & Chernikov, 2014).

Amplitude and intensity simulations for all photon energies considered in this work were performed in the far-field at a distance of 30 m from the source which is corresponding to Fresnel numbers given in Table S1. For a defined number of electrons (see Table S1) their corresponding amplitudes were stored in the matrix for further analysis of coherence functions. The resulting intensity was determined as a sum of intensities of each electron. Angular divergence of the source was determined as the variance value of the intensity

[*] corresponding author: Ivan.Vartaniants@desy.de

distribution at the far field position (Eq. (34)). Transverse positional distribution at the source position was obtained by taking the Fourier transform of the angular field distribution in the far field. The size of the source was determined as the variance value of the intensity distribution at the source position in the middle of the undulator (Eq. (34)). Integration in Eqs. (34) was performed over the finite area defined as a size of the detector in the far-field or size of the source at the source position. In both cases the area corresponded to 95% of the total intensity. Total photon emittance was calculated as the product of the source size and source divergence (Eq. 28) (see Fig. 1 (triangles)). The error of the performed simulation with the XRT software for each natural electron emittance value was calculated as the standard deviation of the corresponding value.

*b) Coherent-mode representation*

To determine independent coherent modes of radiation or perform coherent mode decomposition one needs to solve Fredholm integral equation according to Eq. (10). We performed mode decomposition of the CSD according to Eqs. (9-11) using the XRT software. In the XRT software, CSD is calculated according to Eqs. (7, 8) for the radiation in the far field region for a single electron. The CSD of radiation is, in general, a four-dimensional (4D) matrix (the CSD function depends on coordinates $x_1$, $y_1$; $x_2$, $y_2$). Effectively in the XRT software, one more dimension is added by including a number of electrons so that the correlation function is saved for each electron, which gives additional dimension. The next step is to compose the 4D CSD matrix from the 5D field distribution and to solve the CSD for eigenvalues and eigenfunctions.

The eigenvalue problem for the CSD matrix in XRT software is reduced to two-dimensional (2D) by applying principal component analysis (PCA) (see Fig. S1). After the diagonalization of this matrix, the eigenvalues (mode weights) and eigenfunctions (modes) of CSD were determined (see Fig. 3). If the mode weights are known than the global degree of coherence and the coherent fraction of radiation may be calculated according to Eqs. (12) and (13) (see Fig. 5 (triangles)). It should be also noted, that the global degree of coherence according to the PCA method can be obtained without solving this huge eigenvalue problem. The global degree of coherence, in this case, can be calculated as the ratio between traces of

the CSD matrix $\zeta^{\mathrm{DC}} = \mathrm{Tr}(\mathrm{W}_{\mathrm{PCA}}^2)/\left(\mathrm{Tr}(\mathrm{W}_{\mathrm{PCA}})\right)^2$ , which is equivalent to Eq. (12), where $\mathrm{W}_{\mathrm{PCA}}$ is the rearranged CSD matrix according to the PCA method.

The number of electrons used in the coherent-mode simulations is given in Table S1. The first four coherent modes and their normalized weights for high photon energies of 24 keV and 50 keV for different energy spread values are shown in Fig. S2. The behavior of these modes is similar to the ones observed at 500 eV and 12 keV case (see Fig. 3). The values of the global degree of coherence are given in Table 4.

*c) Degree of transverse coherence*

Simulations of amplitudes and correlation functions in one transverse direction were performed by XRT software as well. One dimensional CSD function at given photon energy was simulated in the far field region as average over an ensemble of electrons according to Eq. (8) in the horizontal direction. Parameters of the simulation are given in Table 5. Results of simulations for high photon energies of 24 keV and 50 keV are shown in Fig. S3 and Fig. S4. As it is seen from these figures, energy spread enlarges rms values $\sigma$ of the intensity distribution (calculated as the variance value, according to Eq. (34)) (Fig. S3-S4 (a,b,c)), while it narrows the CSD and the spectral degree of coherence in anti-diagonal direction (Fig. S3-S4(d-i)), which leads to a decrease of coherence. The values of the degree of transverse coherence in these cases are given in Table 5.

**S2. Details of analytical simulations**

*a) Field amplitude simulation*

In the frame of the analytical approach, source properties were calculated as well (Geloni *et al.*, 2015; Geloni, 2018). In this case, the amplitude $E\left(\theta_x, \theta_y\right)$ in the far field region from a single electron was calculated according to Eq. (33). Total amplitude in the far field region for the electron bunch was defined as

$$E_{total}(\theta_x, \theta_y) = \int E\left(\theta_x, \theta_y\right) f_{\eta_x}(\eta_x) f_{\eta_y}(\eta_y) f_{l_x}(l_x) f_{l_y}(l_y) f_{\gamma_E}(\gamma_E) d\eta_x d\eta_y dl_x dl_y d\gamma_E \,, \tag{1}$$

with

$$f_{\eta_x}(\eta_x) = \frac{1}{\sigma_e'\sqrt{2\pi}} e^{-\frac{\eta_x{}^2}{2\sigma_e'{}^2}}, \qquad f_{\eta_y}(\eta_y) = \frac{1}{\sigma_e'\sqrt{2\pi}} e^{-\frac{\eta_y{}^2}{2\sigma_e'{}^2}}, \tag{2}$$

$$f_{l_x}(l_x) = \frac{1}{\sigma_e\sqrt{2\pi}} e^{-\frac{l_x{}^2}{2\sigma_e{}^2}}, \qquad f_{l_y}(l_y) = \frac{1}{\sigma_e\sqrt{2\pi}} e^{-\frac{l_y{}^2}{2\sigma_e{}^2}}, \tag{3}$$

$$f_{\gamma_E}(\gamma_E) = \frac{1}{\sigma_\gamma\sqrt{2\pi}} e^{-\frac{\gamma_E{}^2}{2\sigma_\gamma{}^2}}, \tag{4}$$

where $f_{\eta_x}(\eta_x)$, $f_{\eta_y}(\eta_y)$ are the angular offset distributions, $f_{l_x}(l_x)$, $f_{l_y}(l_y)$ are the coordinate offset distributions from the undulator axis and $f_{\gamma_E}(\gamma_E)$ is the electron energy distribution according to given energy spread. Effectively it means that each electron got individual random angular and coordinate offsets within the emittance distribution, and random shift to gamma Lorentz factor within the energy spread similar to XRT simulations. Additionally, the 2D amplitude distribution was saved for each electron into 3D matrix, where the third dimension is connected to the number of electrons. The amplitude at the source position $E_0(x, y)$ (in the middle of an undulator) was obtained by the use of the propagator, according to Eq. (33) in the main text.

*b) Source parameters simulation*

Calculations of source parameters were done for zero, $1 \cdot 10^{-3}$, and $2 \cdot 10^{-3}$ relative energy spread values. Amplitude and intensity distribution in the far field region at a distance of 30 m from the source for different photon energies were simulated on the same angular mesh, as in the case of XRT simulations (see Table S1). Total photon emittance was calculated in the same way as it was done previously according to Eq. (34) and Eq. (28) of the main text (see Fig. 1 (dots)). Due to the narrowing of divergence of the radiation higher is the photon energy more electrons were needed to sample the radiation on the virtual detector in order to obtain uniform intensity distribution. In order to accumulate good statistics and get a uniform intensity distribution number of electrons used in this calculation was increased (see Table 5). This adjustment is also valid for larger energy spread values.

*c) Coherent-mode representation, coherent fraction and degree of transverse coherence.*

The same parameters of the analytical simulations of the CSD function were used for XRT simulations (see Table S1). Correlation functions were calculated and saved for each electron according to Eq. (8), with the amplitude distribution calculated according to Eq.

(32). The eigenvalue problem for the 5D CSD matrix was also reduced to two-dimensional by applying PCA as it was done previously by XRT software. In this case, a coherent fraction of the radiation as a function of electron emittance is shown in Fig. 5 (dots). In order to accumulate good statistics the number of electrons used in this calculation was also increased (see Table S1).

Simulations of the amplitude and CSD were also performed in one transverse direction in the frame of the analytical approach (see Figs. S5-S8). One dimensional amplitude distributions for all photon energies considered in this work are shown in Figs. S5-S8 (a,b,c). One dimensional CSD was calculated in the far field region as average over an ensemble of electrons according to Eq. (8) of the main text in the horizontal direction (see Figs. S5-S8 (d,e,f)). The degree of transverse coherence was calculated according to Eq. (19) of the main text. As it is seen from these figures effect of energy spread again enlarges rms values $\sigma$ of the intensity distribution at the same time it narrows the spectral degree of coherence in anti-diagonal direction, which also leads to a decrease in coherence value as it was already shown by XRT simulations. The values of the degree of transverse coherence obtained in the frame of the analytical approach are listed in Table 4. These results are very close to the results obtained from simulations by XRT software.

Due to the fact that analytical analysis matches very well to the simulation of correlation functions performed by XRT software (see Fig. 6 and Figs. S5-S8), it has been extended to other natural electron emittances in a range from 1 to 100 pm rad. The degree of transverse coherence (Eq. (19) of the main text) calculated in the frame of the analytical approach as a function of natural electron emittance for various relative energy spread values is shown in Fig. S9. We observe the same tendencies for the degree of transverse coherence as for coherent fraction of the radiation shown above in Fig. 2 and Fig. 5. At 500 eV and 12 keV we reach diffraction-limited case already at 10 pm rad and 1 pm rad respectively. However, at 24keV and 50 keV even 1 pm rad electron emittance is not sufficient to reach diffraction limit.

## References


Geloni, G., Serkez, S., Khubbutdinov, R., Kocharyan, V. & Saldin, E. (2018). *J. Synchrotron Rad.*, **25**, 1335-1345.

Geloni, G., Kocharyan, V. & Saldin, E. (2015). *J. Synchrotron Rad.*, **22**, 288-316.

Jackson, J.D. (1962). *Classical electrodynamics*, New York: John Wiley & Sons.

Klementiev, K. & Chernikov, R. (2014). *Powerful scriptable ray tracing package xrt*, Advances in Computational Methods for XRay Optics III, San Diego, pp. 92090-92096. Online documentation at xrt.readthedocs.io; doi:10.5281/zenodo.1252468

Sanchez del Rio, M. (2018). *arXiv:1801.07542v2 [physics.acc-ph]*.

**Table S1.**

Details of XRT simulations and analytical approach. $N_e$ is the number of electrons used in the simulations of photon emittance and simulations of CSD.

| Energy, keV | $N_e$ (photon emittance) | | $N_e$ (coherent-mode decomposition) | | Angular mesh (AxA) | | | Size of the virtual detector, mm | Energy spread, $10^{-3}$ | Fresnel number |
|---|---|---|---|---|---|---|---|---|---|---|
| | | | | | Photon Emittance | Mode decompo-sition | | | | |
| | XRT | Analytical | XRT | Analytical | XRT & Analytical | XRT | Analytical | | | |
| 0.5 | $5 \cdot 10^3$ | $5 \cdot 10^3$ | $5 \cdot 10^3$ | $10^5$ | 512 | 128 | 256 | 5.4 | 0 | 0.05 |
| | | | | | | | | | 1 | 0.05 |
| | | | | | | | | | 2 | 0.06 |
| 12 | $5 \cdot 10^3$ | $10^4$ | $5 \cdot 10^3$ | $10^5$ | 512 | 128 | 256 | 2.4 | 0 | 0.09 |
| | | | | | | | | | 1 | 0.14 |
| | | | | | | | | | 2 | 0.21 |
| 24 | $5 \cdot 10^3$ | $1.5 \cdot 10^4$ | $5 \cdot 10^3$ | $10^5$ | 512 | 128 | 256 | 1.8 | 0 | 0.13 |
| | | | | | | | | | 1 | 0.19 |
| | | | | | | | | | 2 | 0.26 |
| 50 | $5 \cdot 10^3$ | $1.5 \cdot 10^4$ | $5 \cdot 10^3$ | $10^5$ | 512 | 128 | 256 | 1.2 | 0 | 0.19 |
| | | | | | | | | | 1 | 0.3 |
| | | | | | | | | | 2 | 0.44 |

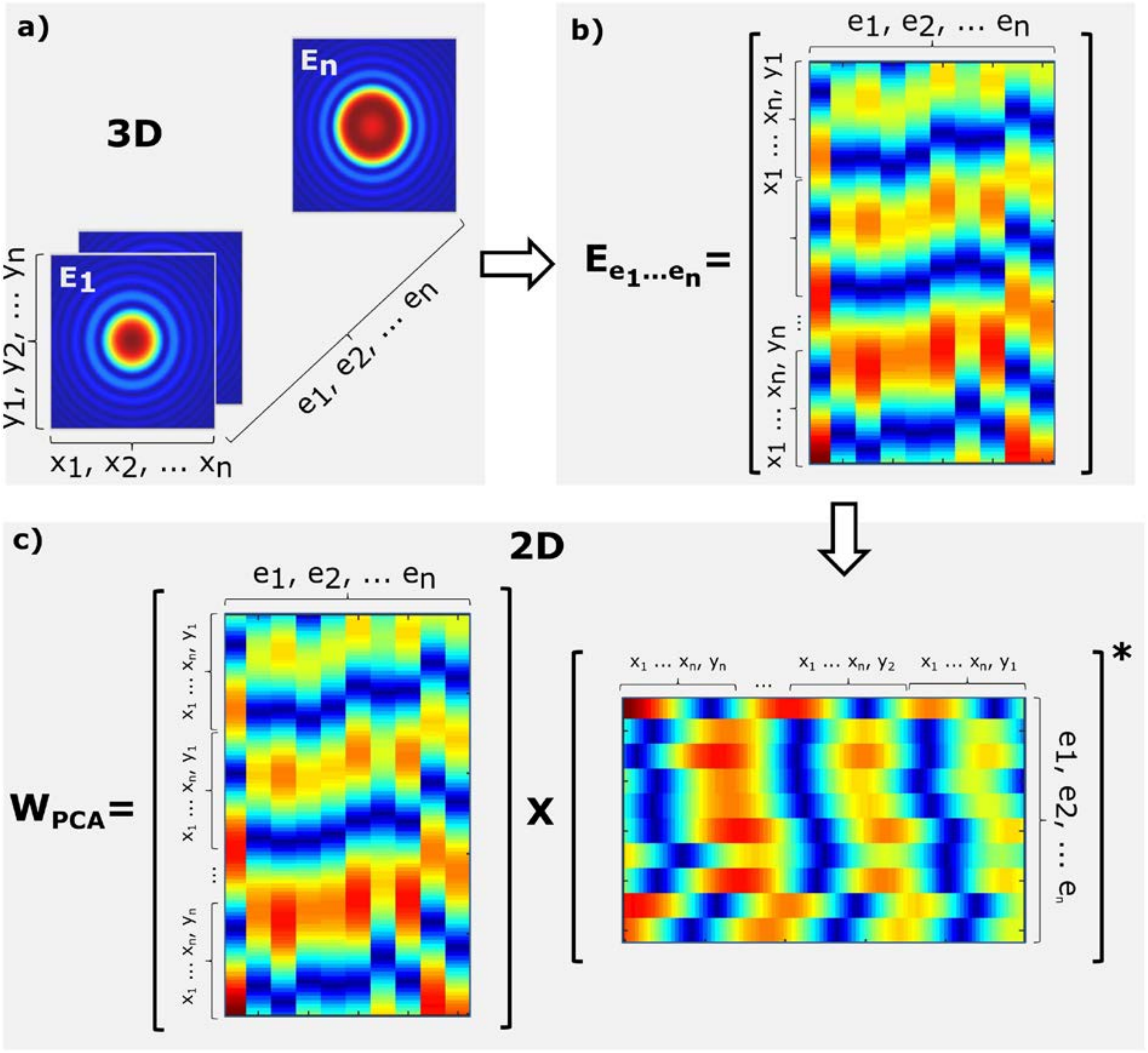


**Figure S1** PCA method used in the coherent mode decomposition. a) 2D amplitudes stored in a 3D matrix for each electron $e_1 \ldots e_n$, where 3rd dimension is connected to the number of electrons. b) 3D matrix of amplitudes, rearranged to 2D matrix $E_{e_1-e_n}$, where each column contains all spatial information about an amplitude corresponding to one of the electrons $e_i$ from the electron bunch. c) Cross-spectral density matrix $W_{PCA}$ obtained by multiplication of matrix $E_{e_1-e_n}$ by its complex conjugated and transposed matrix.

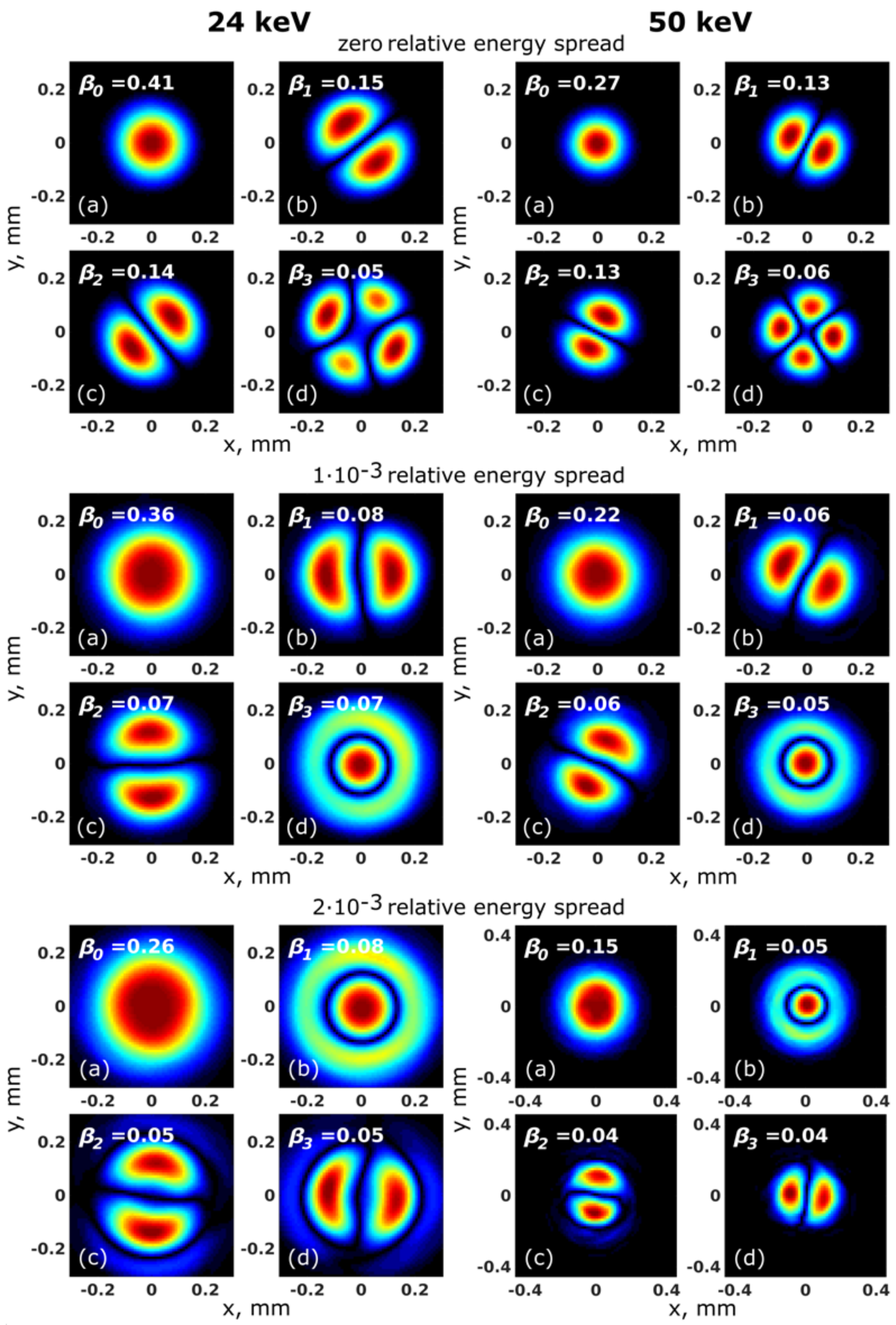


**Figure S2** First four modes and their normalized weights $\beta_j$ obtained from the coherent mode decomposition of the CSD at 24 keV (left column) and 50 keV (right column) photon energy for three different relative energy spread values obtained by XRT simulations.

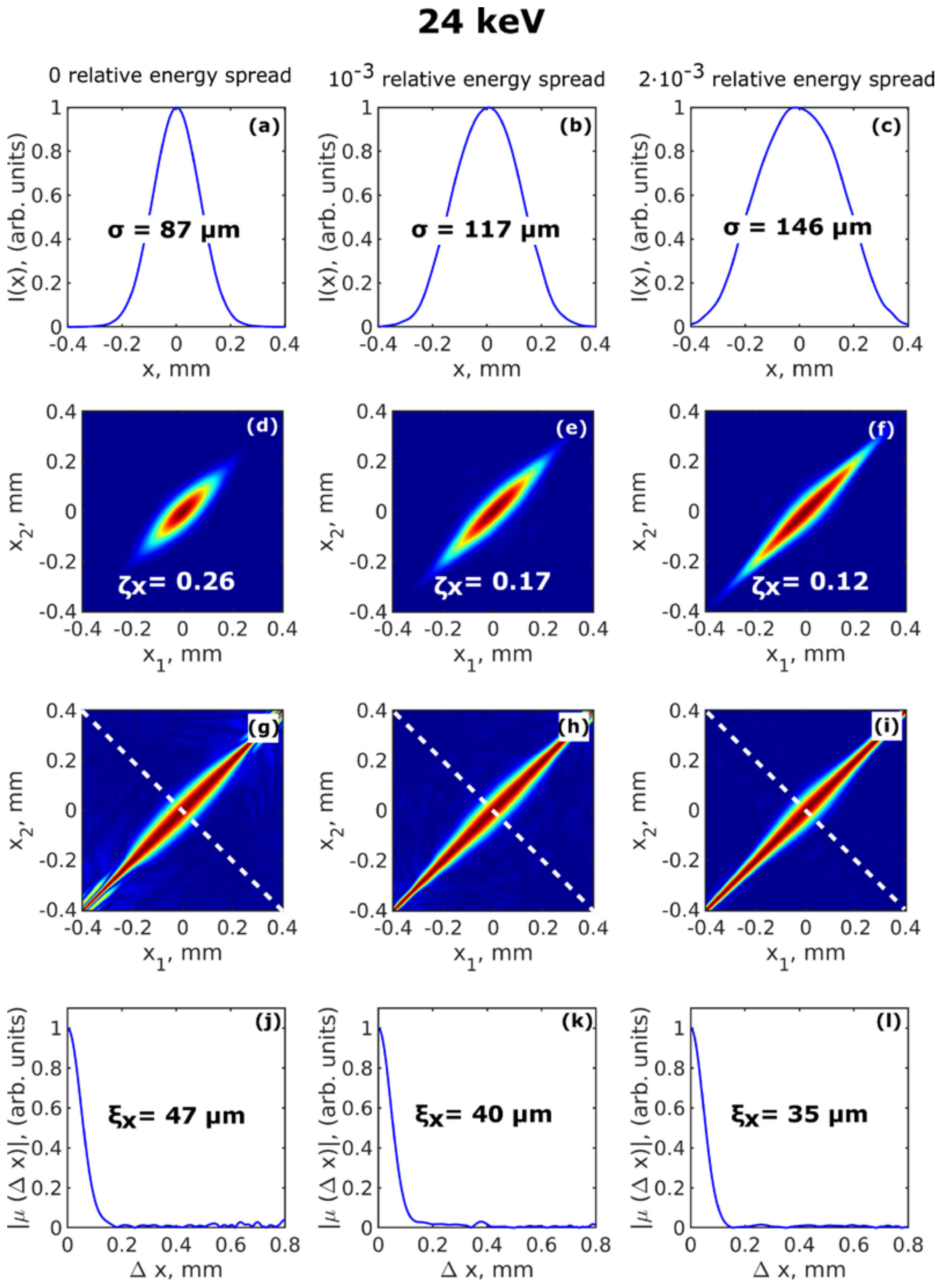


**Figure S3** Simulations of the correlation functions in horizontal direction performed by the XRT software for 24 keV photon energy and different energy spread values. Intensity distribution I($x$) (a-c), absolute value of the CSD in the horizontal direction |W($x_1$,$x_2$)| (d-f), absolute value of the SDC |μ($x_1$,$x_2$)| (g-i), and absolute value of the SDC along the anti-diagonal line (shown in (g-i)) as a function of separation of two points |μ($\Delta x$)| (j-l) simulated in horizontal direction 30 m downstream from the undulator source. In (a-c) $\sigma$ is the rms value of the beam size, in (d-f) $\zeta_x$ is the transverse degree of coherence, in (j-l) $\xi_x$ is the coherence length determined in the horizontal direction.

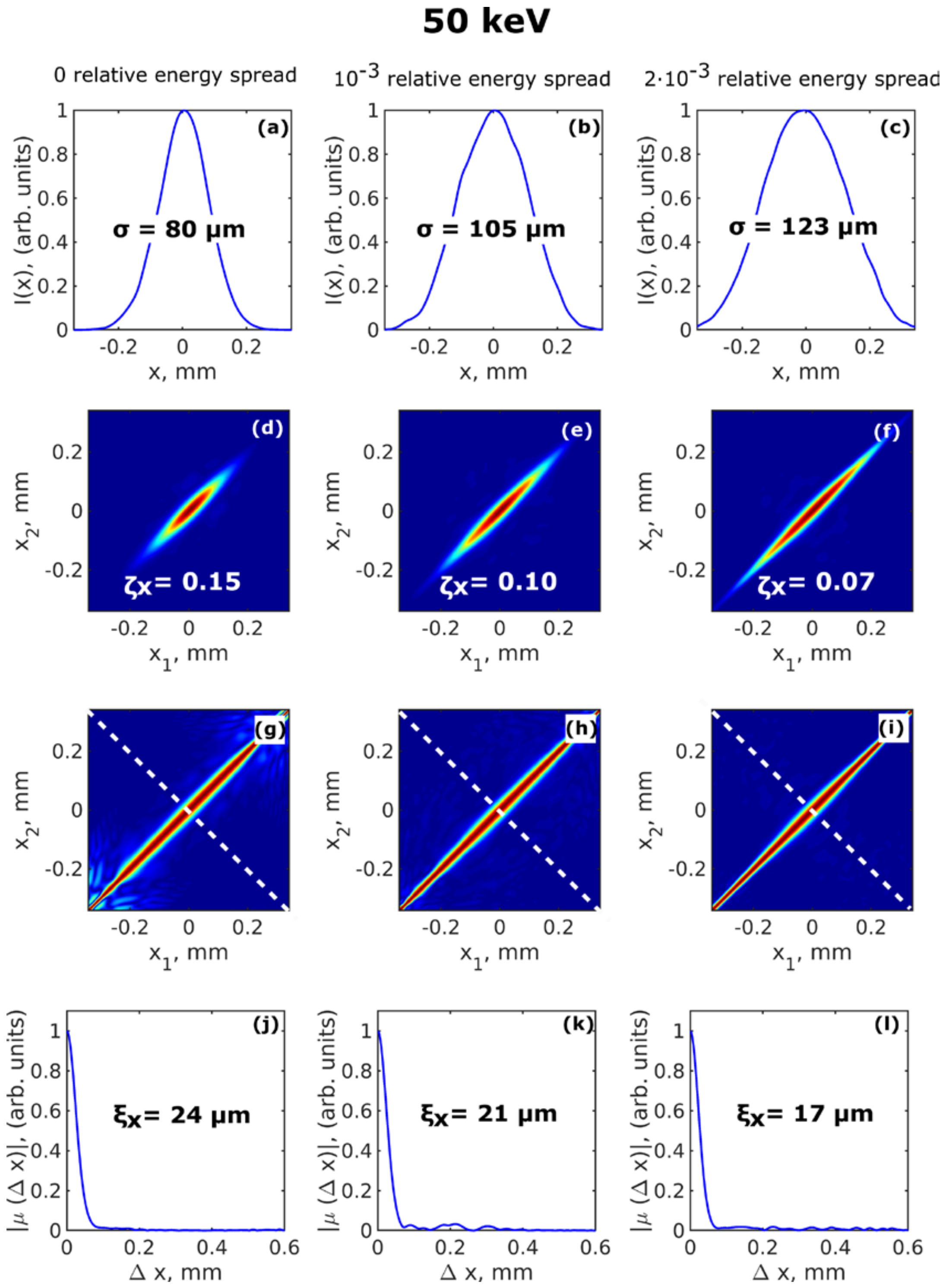


**Figure S4** Simulations of the correlation functions in horizontal direction performed by the XRT software for 50 keV photon energy and different energy spread values. Intensity distribution I($x$) (a-c), absolute value of the CSD in the horizontal direction |W($x_1$,$x_2$)| (d-f), absolute value of the SDC |μ($x_1$,$x_2$)| (g-i), and absolute value of the SDC along the anti-diagonal line (shown in (g-i)) as a function of separation of two points |μ($\Delta x$)| (j-l) simulated in horizontal direction 30 m downstream from the undulator source. In (a-c) $\sigma$ is the rms value of the beam size, in (d-f) $\zeta_x$ is the transverse degree of coherence, in (j-l) $\xi_x$ is the coherence length determined in the horizontal direction.

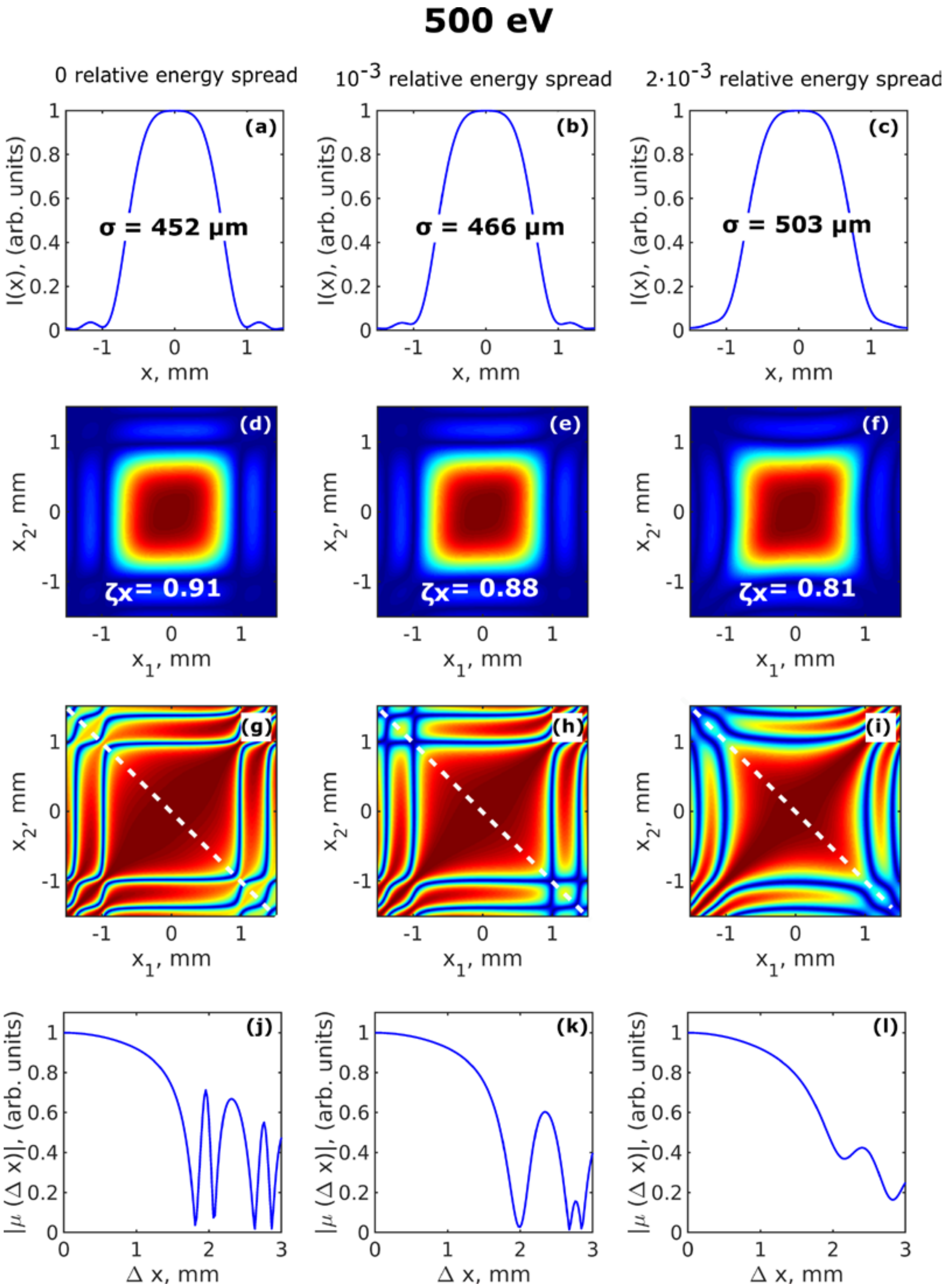


**Figure S5** Simulations of the correlation functions in horizontal direction performed by the analytical approach for 500 eV photon energy and different energy spread values. (a-c) Intensity distribution $I(x)$, (d-f) absolute value of the CSD in the horizontal direction $|W(x_1,x_2)|$, (g-i) absolute value of the SDC $|\mu(x_1,x_2)|$, (j-l) absolute value of the SDC along the anti-diagonal line (shown in (g-i)) as a function of separation of two points $|\mu(\Delta x)|$ simulated in horizontal direction 30 m downstream from the undulator source. In (a-c) $\sigma$ is the rms value of the beam size, in (d-f) $\zeta_x$ is the transverse degree of coherence, in (j-l) $\xi_x$ is the coherence length determined in the horizontal direction.

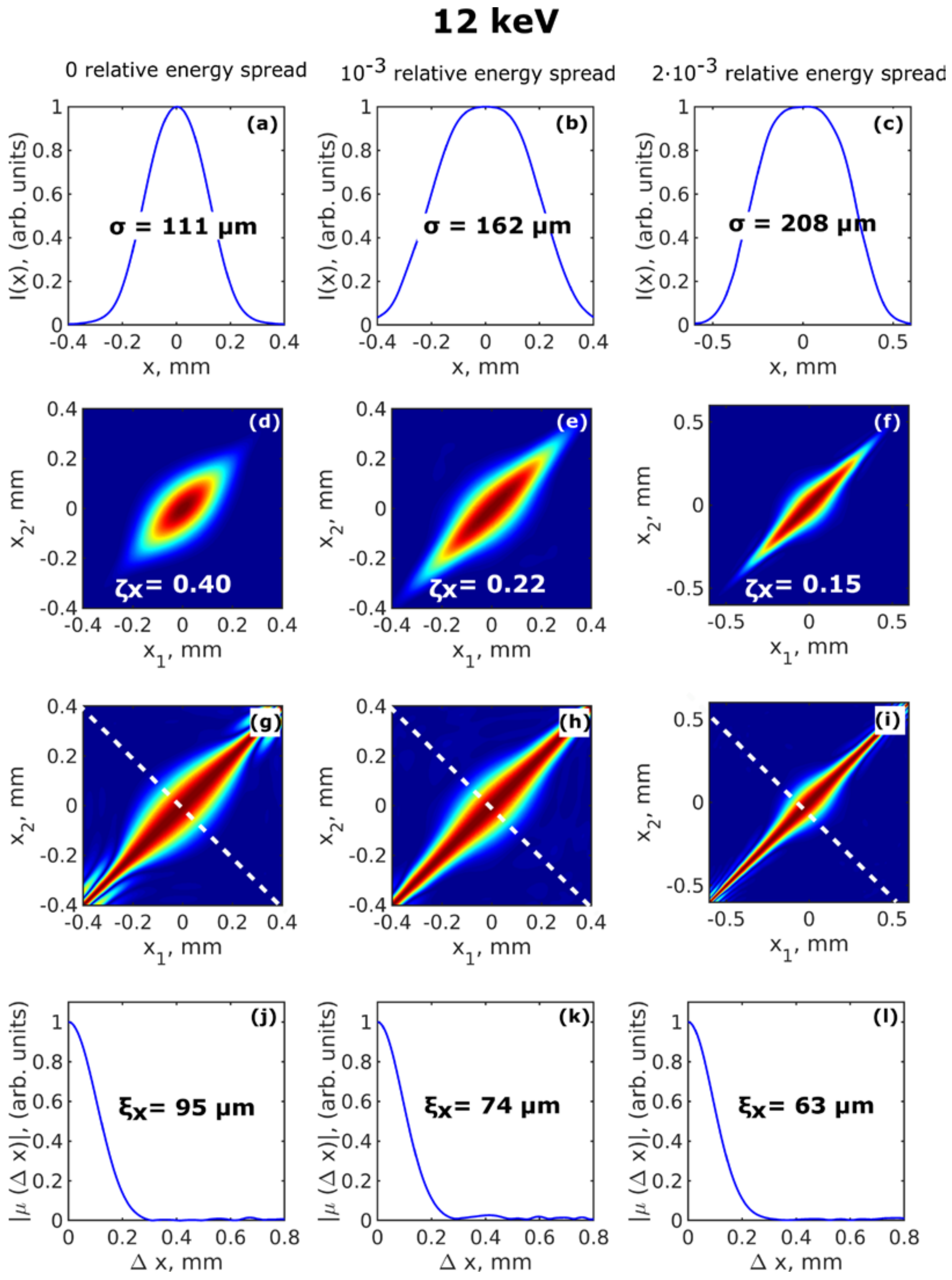


**Figure S6** Simulations of the correlation functions in horizontal direction performed by the analytical approach for 12 keV photon energy and different energy spread values. (a-c) Intensity distribution $I(x)$, (d-f) absolute value of the CSD in the horizontal direction $|W(x_1,x_2)|$, (g-i) absolute value of the SDC $|\mu(x_1,x_2)|$, (j-l) absolute value of the SDC along the anti-diagonal line (shown in (g-i)) as a function of separation of two points $|\mu(\Delta x)|$ simulated in horizontal direction 30 m downstream from the undulator source. In (a-c) $\sigma$ is the rms value of the beam size, in (d-f) $\zeta_x$ is the transverse degree of coherence, in (j-l) $\xi_x$ is the coherence length determined in the horizontal direction.

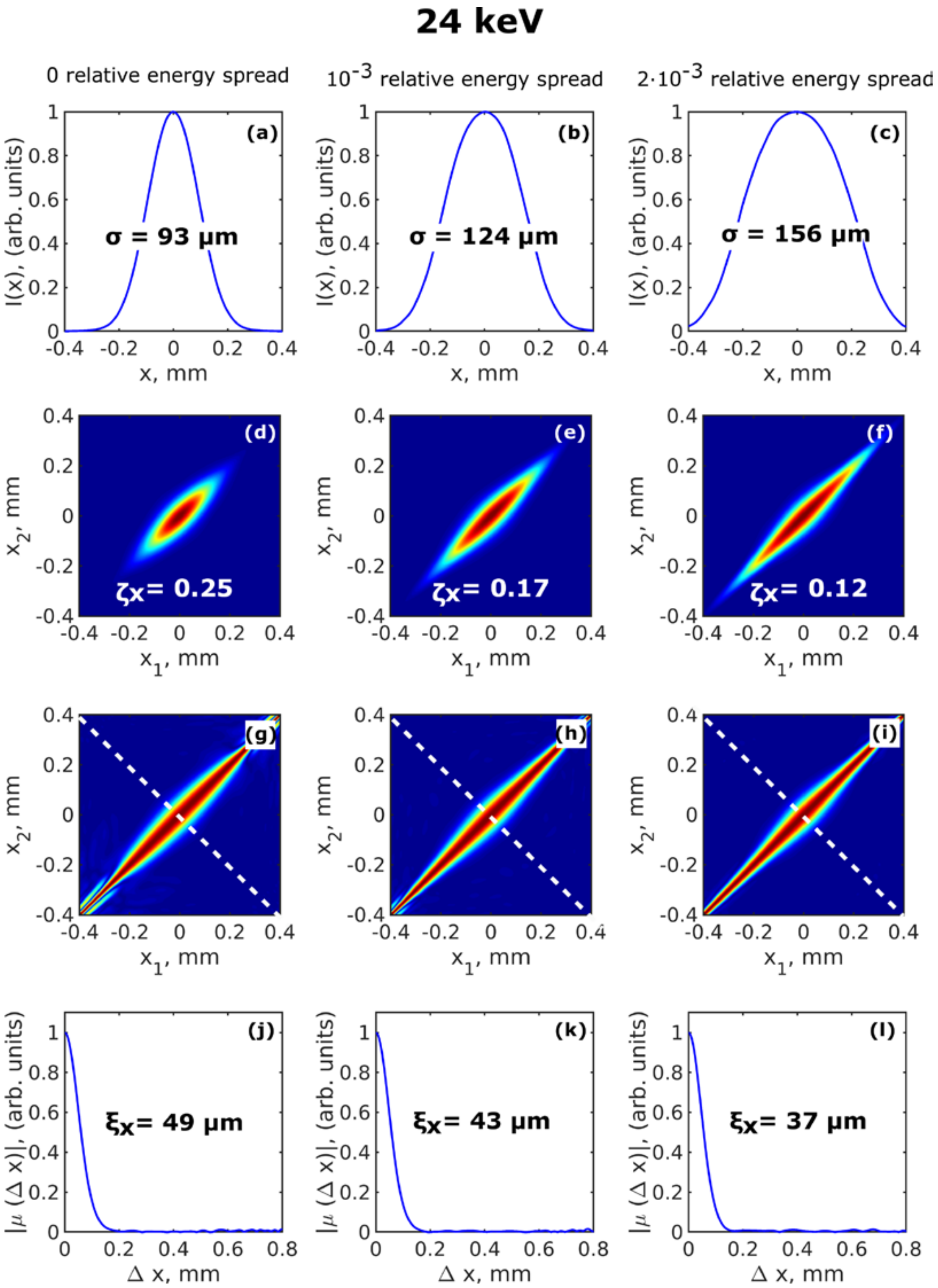


**Figure S7** Simulations of the correlation functions in horizontal direction performed by the analytical approach for 24 keV photon energy and different energy spread values. (a-c) Intensity distribution $I(x)$, (d-f) absolute value of the CSD in the horizontal direction $|W(x_1,x_2)|$, (g-i) absolute value of the SDC $|\mu(x_1,x_2)|$, (j-l) absolute value of the SDC along the anti-diagonal line (shown in (g-i)) as a function of separation of two points $|\mu(\Delta x)|$ simulated in horizontal direction 30 m downstream from the undulator source. In (a-c) $\sigma$ is the rms value of the beam size, in (d-f) $\zeta_x$ is the transverse degree of coherence, in (j-l) $\xi_x$ is the coherence length determined in the horizontal direction.

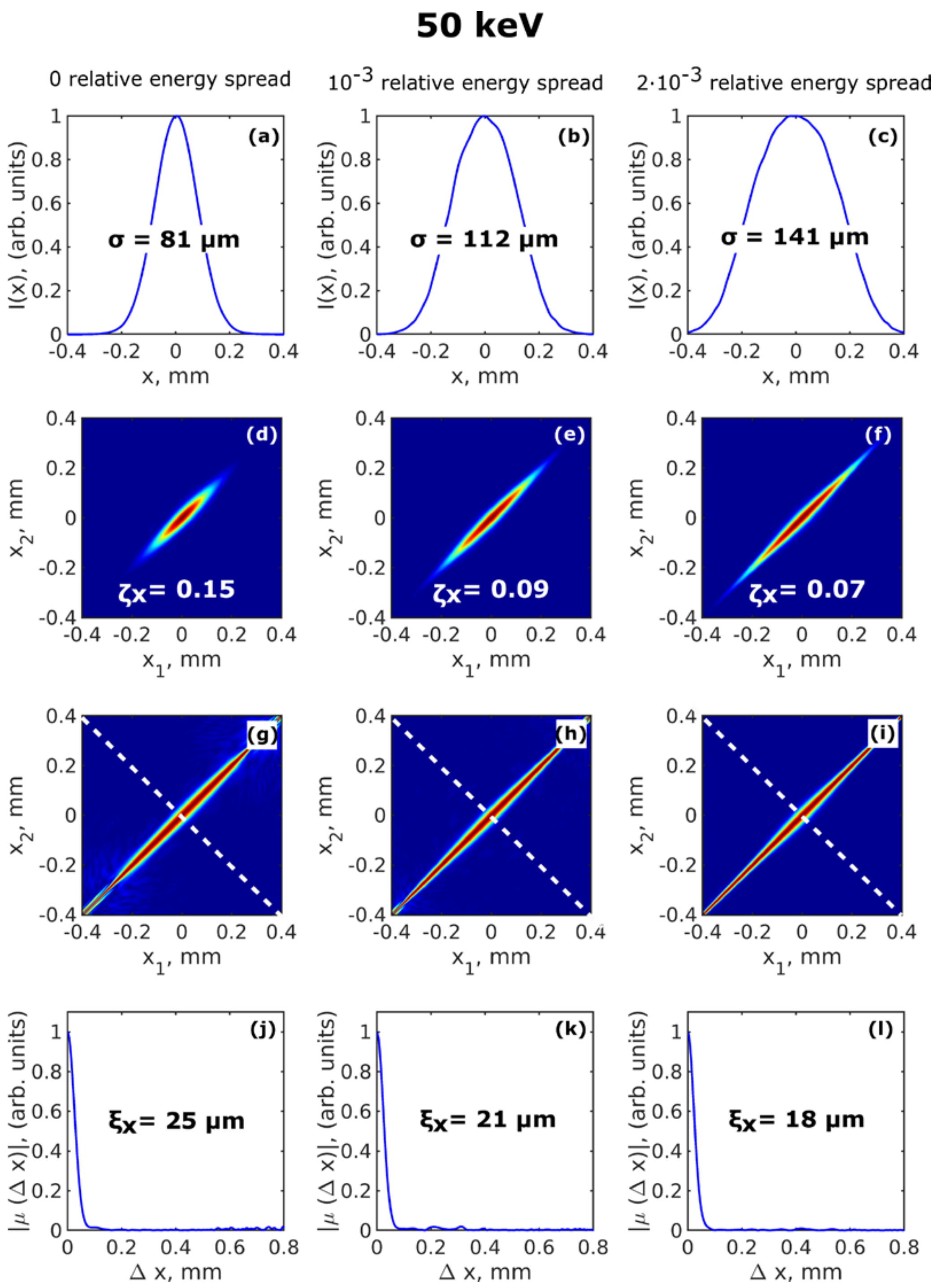


**Figure S8** Simulations of the correlation functions in horizontal direction performed by the analytical approach for 50 keV photon energy and different energy spread values. (a-c) Intensity distribution $I(x)$, (d-f) absolute value of the CSD in the horizontal direction $|W(x_1,x_2)|$, (g-i) absolute value of the SDC $|\mu(x_1,x_2)|$, (j-l) absolute value of the SDC along the anti-diagonal line (shown in (g-i)) as a function of separation of two points $|\mu(\Delta x)|$ simulated in horizontal direction 30 m downstream from the undulator source. In (a-c) $\sigma$ is the rms value of the beam size, in (d-f) $\zeta_x$ is the transverse degree of coherence, in (j-l) $\xi_x$ is the coherence length determined in the horizontal direction.

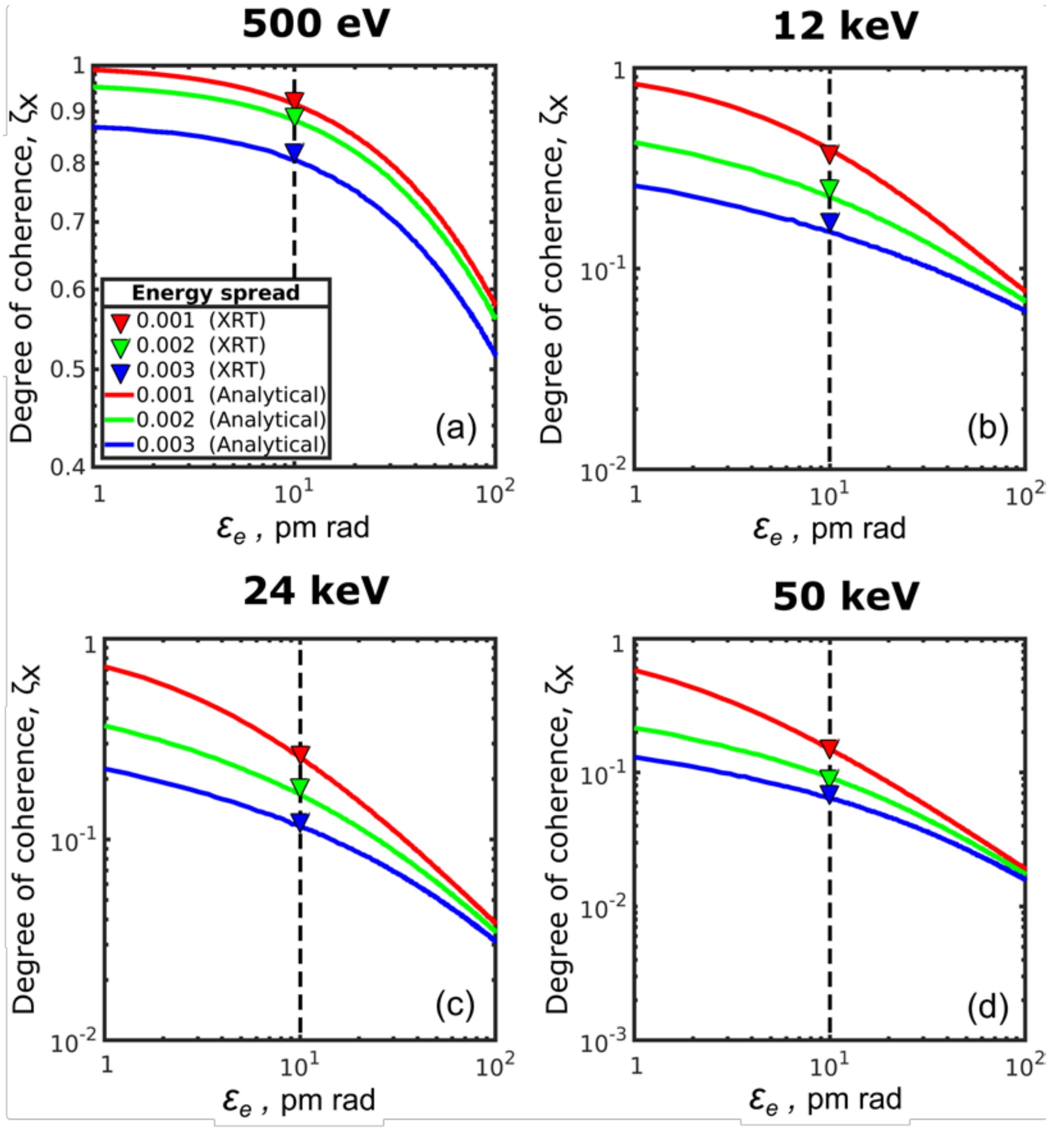


**Figure S9** Simulation of the degree of transverse coherence $\zeta_x$ obtained from XRT and analytical analysis (Eq. (5)) as a function of natural electron emittance for 500 eV, 12 keV, 24keV, and 50 keV.
